# Songorine modulates macrophage polarization and metabolic reprogramming to alleviate inflammation in osteoarthritis

**DOI:** 10.3389/fimmu.2024.1344949

**Published:** 2024-02-13

**Authors:** Xi-Xi He, Yuan-Jun Huang, Chun-Long Hu, Qiong-Qian Xu, Qing-Jun Wei

**Affiliations:** ^1^ Department of Orthopedics Trauma and Hand Surgery, The First Affiliated Hospital of Guangxi Medical University, Nanning, China; ^2^ Department of Pediatric Surgery, Qilu Hospital of Shandong University, Jinan, China

**Keywords:** osteoarthritis, Songorine, metabolic alteration, oxidative stress, inflammation

## Abstract

**Introduction:**

Osteoarthritis (OA) is a prevalent joint disorder characterized by multifaceted pathogenesis, with macrophage dysregulation playing a critical role in perpetuating inflammation and joint degeneration.

**Methods:**

This study focuses on Songorine, derived from Aconitum soongaricum Stapf, aiming to unravel its therapeutic mechanisms in OA. Comprehensive analyses, including PCR, Western blot, and immunofluorescence, were employed to evaluate Songorine's impact on the joint microenvironment and macrophage polarization. RNA-seq analysis was conducted to unravel its anti-inflammatory mechanisms in macrophages. Metabolic alterations were explored through extracellular acidification rate monitoring, molecular docking simulations, and PCR assays. Oxygen consumption rate measurements were used to assess mitochondrial oxidative phosphorylation, and Songorine's influence on macrophage oxidative stress was evaluated through gene expression and ROS assays.

**Results:**

Songorine effectively shifted macrophage polarization from a pro-inflammatory M1 phenotype to an anti-inflammatory M2 phenotype. Notably, Songorine induced metabolic reprogramming, inhibiting glycolysis and promoting mitochondrial oxidative phosphorylation. This metabolic shift correlated with a reduction in macrophage oxidative stress, highlighting Songorine's potential as an oxidative stress inhibitor.

**Discussion:**

In an *in vivo* rat model of OA, Songorine exhibited protective effects against cartilage damage and synovial inflammation, emphasizing its therapeutic potential. This comprehensive study elucidates Songorine's multifaceted impact on macrophage modulation, metabolic reprogramming, and the inflammatory microenvironment, providing a theoretical foundation for its therapeutic potential in OA.

## Graphical Abstract

The signaling pathways through which Songorine binds to metabolic pathway, resulting in metabolic reprogramming, polarization of macrophages, inhibition of inflammatory factors secretion, regulation of the cartilage anabolism, and protection against OA.

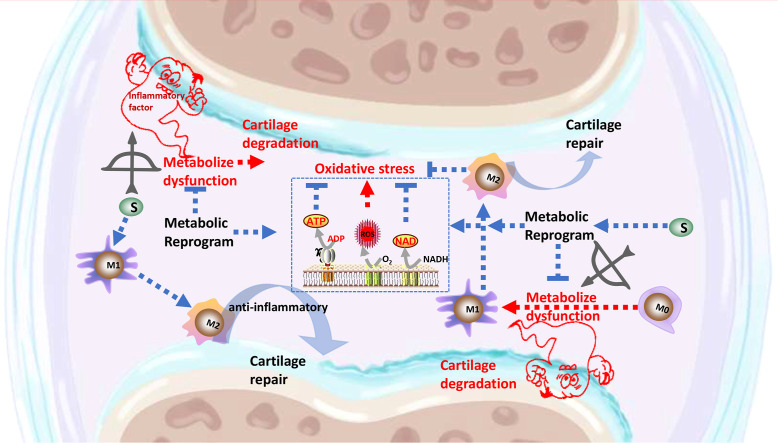

## 1 Introduction

Osteoarthritis (OA) stands as a prevalent and debilitating joint disorder characterized by the progressive deterioration of articular cartilage, subchondral bone alterations, and the formation of osteophytes (1, 2). With a rising global incidence, particularly in the aging population, OA significantly impacts the quality of life for affected individuals. The intricate interplay of genetic, mechanical, and biochemical factors contributes to the multifaceted pathogenesis of OA. Recent research has unveiled the crucial role of inflammatory processes and immune cell involvement, particularly macrophages, in driving the structural changes observed in OA joints (3, 4).

Macrophages, as key players in the immune system, exhibit a remarkable plasticity, adopting distinct phenotypes in response to environmental cues. Classically activated M1 macrophages contribute to inflammation and tissue degradation, while alternatively activated M2 macrophages are associated with tissue repair and anti-inflammatory responses. The dysregulation of macrophage polarization has emerged as a central player in the perpetuation of OA, offering a promising avenue for therapeutic intervention (5, 6). The specific dysregulation of macrophage polarization is recognized as a pivotal factor in the context of OA. Dysregulated macrophages contribute to the perpetuation of inflammation, tissue degradation, and altered repair mechanisms within the affected joints. The intricate balance between M1 and M2 macrophage phenotypes becomes disrupted, leading to an environment conducive to OA progression. Understanding this dysregulation provides a crucial foundation for exploring targeted therapeutic interventions that address the nuanced role of macrophages in OA.

The immense potential of natural products in treating various diseases, including OA, has been a continuous exploration. Notably, key findings from current research underscore the promising role of natural products in mitigating OA symptoms, potentially influencing macrophage behavior and inflammatory responses within the joint microenvironment (7, 8). Crucially, alkaloids, widespread in nature (9), play a key role in Traditional Chinese Medicine’s therapeutic effects, exhibiting significant anti-cancer, anti-inflammatory, and antioxidant activities (10–12). In this context, Aconitum, commonly known as “wutou,” stands out as a repository of approximately 450 alkaloids and has been extensively utilized for treating various diseases in China, Japan, and other regions (13, 14). Aconitum has been traditionally employed to alleviate a spectrum of ailments, including inflammatory conditions, cardiovascular disorders, and nervous system disturbances (15). Aconitum soongaricum Stapf, a specific species within the Aconitum genus, is particularly noteworthy for housing Songorine, a C20-diterpenoid alkaloid with a range of characteristics, including anti-inflammatory, antiarrhythmic, and anti-central nervous system disturbance properties (16–18). This convergence of traditional wisdom, scientific exploration, and the identification of specific bioactive compounds like Songorine underscores the intricate interplay within Traditional Chinese Medicine and its potential in addressing complex diseases such as OA.

Despite these advancements, there exists a critical need to consolidate and synthesize key findings from ongoing research on natural products in OA. A comprehensive understanding of the therapeutic potential of these natural products, especially in the context of immune cell modulation and macrophage behavior, will contribute to a more holistic approach in developing targeted and effective therapies for OA.

Therefore, our study focuses on the natural compound Songorine, and its potential therapeutic role in OA. Through a comprehensive exploration of macrophage modulation, metabolic reprogramming, and the impact on the inflammatory microenvironment, we aim to unravel the molecular mechanisms that underlie Songorine’s effects in the context of OA. This research endeavors to address the critical gap in understanding how Songorine, a specific bioactive compound from Aconitum soongaricum Stapf, influences macrophage behavior and immune responses in the OA microenvironment. we aim to contribute valuable insights that will inform the development of nuanced and effective therapeutic strategies for OA, considering the complex interplay between inflammation, immune responses, and joint degeneration.

## 2 Materials and methods

### 2.1 Cell culture

The murine macrophages (RAW 264.7) from Procell Life Science & Technology Co., Ltd. (Hubei, China) were incubated in medium at a temperature of 37°C in a moist environment with 5% CO2. Songorine (purity is 99.48% by high performance liquid chromatography with diode array detector, 230nm) obtained from Chengdu Biopurify Phytochemicals Ltd. (Cat. BP029) was used in this study. and used as a stimulator for macrophage activation. M1 polarization was induced by treating RAW 264.7 cells with Lipopolysaccharides (LPS) From Escherichia coli 055:B5 (Solarbio, Cat. L8880, The potency of LPS is greater than or equal to 500,000 EU per mg, < 1% protein) at a concentration of 100 ng/mL for a duration of 24 hours.

In order to assess the impacts and mechanisms of Songorine on the immune system, the conditioned media obtained from Songorine-treated macrophages were gathered and utilized for the cultivation of chondrocytes. The control group consisted of chondrocytes that were not treated with Songorine. The M0CM group was designated as the chondrocytes that were cultured in a conditioned medium derived from M0 macrophages. M1CM refers to chondrocytes that were cultured in a conditioned medium derived from M1 macrophages. S10CM and S50CM were designated as chondrocytes that were cultured in a conditioned medium obtained from M1 macrophages that had been treated with varying concentrations of Songorine for a duration of 24 hours. Following a 24-hour incubation period, the chondrocytes were gathered for additional examination.

### 2.2 Identification of reactive oxygen species

M0 macrophages were placed in 6-well dishes and permitted to proliferate until reaching 60–70% confluence. M0 macrophages were stimulated with LPS to induce M1 polarization. Afterwards, the cells were cultured with either 10 or 50 μM Songorine. Following the manufacturer’s guidelines of a ROS Assay Kit (Beyotime, Cat.S0033S) (19), the cells were washed three times with PBS and incubated with the ROS probe after 24 hours of treatment. Following an additional PBS wash, the specimens were examined and captured using a fluorescence microscope (Olympus, Japan). The fluorescence intensities of the images were quantified using the Image J software. Chondrocytes were placed in 6-well dishes and grown in conditioned media (M0CM, M1CM, S10CM, and S50CM) for 24 hours. The following procedures remained unchanged as described above, including washing with PBS, incubating with the ROS probe, and imaging and quantifying fluorescence intensity using Image J software.

### 2.3 Analysis of quantitative real-time PCR

Total mRNA was extracted using RNAfast200 (Fastagen, China) and then converted into cDNA using a reverse transcription kit (TOYOBO, Cat. FSQ-101), according to the manufacturer’s instructions. In order to examine the levels of gene expression, the qRT-PCR analysis provided by the Kit (TAKARA, Cat. RR820A). Internal controls in the form of GAPDH and actin mRNA as housekeeping genes were employed. **Table 1** contains the list of primers utilized for the target genes.

**Table 1:** The primers for the target genes.

Target Gene	Forward primer sequence	Forward primer sequence
PFKFB3	GGAGTCCGCAAAACAGGATG	GATGCGAGGCTTTTTGGTGG
GAPDH (Rat)	TCTCTGCTCCTCCCTGTTCT	ATCCGTTCACACCGACCTTC
GAPDH (Mus)	TGGATTTGGACGCATTGGTC	TTTGCACTGGTACGTGTTGAT
β-actin	GAGCTACGAGCTGCCTGACG	CCTAGAAGCATTTGCGGTGG
HK-II	GGTGCTGTGGCGAATCAAAG	GAGACGCTTGGCAAAATGGG
GLUT	TTAATCGCTTTGGCAGGCGG	GTCAGGCCACAGTACACTCC
LDHA	GAGCCACTGTCGCCGATCTC	AATCTTTTGGGACCCTGCACC
ACAN	CCTGCTACTTCATCGACCCC	AGATGCTGTTGACTCGAACCT
SOX9	TCCAGCAAGAACAAGCCACA	CGAAGGGTCTCTTCTCGCTC
COL-2α	GTCCTACAATGTCAGGGCCA	ACCCCTCTCTCCCTTGTCAC
IL-1β (Rat)	GCACAGTTCCCCAACTGGTA	GGAGACTGCCCATTCTCGAC
IL-6 (Rat)	ACAAGTCCGGAGAGGAGACT	ACAGTGCATCATCGCTGTTC
MMP-13	GGACAAAGACTATCCCCGCC	GGCATGACTCTCACAATGCG
CD206	GCACTGGGTTGCATTGGTTT	CCTGAGTGGCTTACGTGGTT
CD86	AACTTACGGAAGCACCCACG	ATAAGCTTGCGTCTCCACGG
IL-10	CCTCGTTTGTACCTCTCTCCG	AGGACACCATAGCAAAGGGC
iNOS	GGGTCACAACTTTACAGGGAGT	GAGTGAACAAGACCCAAGCG
IL-1β (Mus)	TGCCACCTTTTGACAGTGATG	TGTGCTGCTGCGAGATTTGA
IL-6 (Mus)	CAACGATGATGCACTTGCAGA	TGACTCCAGCTTATCTCTTGGT

### 2.4 Simple western (Jess by ProteinSimple)

We used a Simple Western blot technique to examine the anti-inflammatory mechanism and the impact of Songorine on cellular metabolism. The cells were placed in 6-well dishes and incubated for 24 hours until they achieved a 70% confluence. Afterwards, the cells were exposed to inflammatory agents (LPS) for a duration of 24 hours. Subsequent to the stimulation, the cells were subjected to various doses of Songorine for another 24-hour period.

Following different procedures, the cells were lysed by radioimmunoprecipitation (RIPA) buffer (Boster, China, Cat. AR0102S) supplemented with 1 mmol/L phenylmethylsulfonyl fluoride (PMSF, Boster, China, Cat. AR0102S). The lysis process involved placing the samples on ice and vortexing every 5 minutes for a total duration of 30 minutes. Following lysis, the cellular extracts underwent centrifugation at 12,000×g for 15 minutes at 4°C. Subsequently, the protein concentrations in the resulting supernatants were quantified using a BCA protein assay kit (Boster, China, Cat. AR0197). To ensure accurate measurements, the ultimate protein concentration of every sample was modified to 0.2 μg/μL. The protein samples were subjected to a temperature of 95°C for a duration of 5 minutes in a 0.1x sample buffer and 5x Master Mix. Subsequently, they were separated through capillary electrophoresis employing the JESS system manufactured in the United States. To identify particular proteins, the main antibodies, such as rabbit anti-IL-1β (1:40), anti-IL-6 (1: 100), mouse anti-CD86 (1:200) were diluted with antibody diluent. Subsequently, 10 μL of streptavidin-HRP and secondary antibodies were added to the wells. Protein expression levels were evaluated using the Compass software for SW 4.1.0, which provided a quantitative analysis of the detected protein bands. The Simple Western blot technique allowed for examination of the anti-inflammatory properties of Songorine and the verification of its impact on cellular metabolism by analyzing the expression levels of specific target proteins.

### 2.5 Immunofluorescence

The protein expression of target genes in the cells was assessed using Immunofluorescence (IF) staining. The IF staining was carried out following the guidelines given with the DyLight 488-SABC Kit (Boster, SA1094). Rabbit anti-IL-1β (1:200; Proteintech, 16806-1-AP), mouse anti-CD86 (1:200; Santa Cruz Biotechnology, sc-28347), and rabbit anti-CD206 (1:200; Proteintech, 18704-1-AP) were the primary antibodies utilized. The antibodies were employed to specifically target and attach to the desired proteins. Following the incubation period with primary antibodies, the cells underwent a washing step to eliminate any antibodies that were not bound. Afterward, fluorophore-conjugated secondary antibodies were employed to visualize the target proteins. A DyLight 488-SABC kit (Boster, SA1094) provided the appropriate secondary antibodies for staining. In the end, cells that were stained with immunofluorescence were observed and captured using a fluorescence microscope. By using suitable fluorescence channels, the captured images enabled the examination and interpretation of IL1, IL-6, CD86, and CD206 protein expression levels in the cells.

### 2.6 Measurement of cellular metabolism

Before the experiment, a hydration plate was prepared by adding 200 μL of sterile water to each well to ensure proper hydration of the sensors on the probe plate. Afterward, the probe plate device was placed in a cell incubator without CO_2_ and incubated at a temperature of 37°C for the duration of the night. The Seahorse XFe96 test system was preheated for at least 5 h at 37°C. Different inflammatory factors were used to stimulate cells, including chondrocytes and macrophages, which were then treated with varying concentrations of Songorine. These cells were plated in Seahorse XF96 plates with a density of 10,000 cells per well. After 24 h of culture, a seahorse XFe96 assay system was performed using the Agilent Seahorse XF analyzer. During the experiment, the growth medium was substituted with Seahorse XF assay solution, and the cells were subsequently placed in a cell incubator devoid of CO_2_ at a temperature of 37°C for a duration of 60 minutes to achieve equilibrium. The glycolytic stress test (103020-100, Agilent Technologies) was conducted using an assay solution containing Seahorse XF DMEM Medium (103575–100), 2mM glutamine (103579–100), pH 7.4. Measurements of the extracellular acidification rate (ECAR) were conducted at intervals of 5 minutes, both prior to and following the consecutive introduction of glucose (10 mM), oligomycin (1 μM), and 2-DG (50 μM). To assess the cellular oxygen consumption rate (OCR), a Mito Stress Test Kit (103015-100, Agilent Technologies) was employed (20). Glucose was present in the assay solution containing Seahorse XF DMEM Medium 97ml (103575–100), 1mL glucose (103577–100), 1mL pyruvate (103578-100), 1mL glutamine (103579-100), pH 7.4, and measurements were conducted at 5-minute intervals prior to and following the consecutive introduction of oligomycin (1.5 μM), FCCP (1 μM), and Rotenone/Antimycin A (0.5 μM) into the injection ports (21).

The collected data were examined utilizing Wave software, which enabled the interpretation and analysis of the horse assay results. This experimental approach allowed for the assessment of glycolytic activity and mitochondrial respiration, providing valuable insights into the metabolic changes induced by different inflammatory stimuli and the effects of Songorine treatment.

The determination of the total amount of oxidized and reduced Nicotinamide adenine dinucleotide (NAD) and the individual amounts of reduced NADH content was conducted through the utilization of the NAD(+)/NADH assay kit employing the WST-8 method (Beyotime, Nantong, China), following the guidelines provided by the manufacturer (22).

### 2.7 The process of RNA sequencing and the subsequent analysis

To explore the impact of LPS on gene expression and the potential regulatory function of Songorine, macrophages were subjected to diverse treatment conditions during culturing. For each type of cell, the experimental setup consisted of three groups (1) cells cultured without any treatment, (2) cells treated solely with LPS (100 ng/mL), and (3) cells treated with 50 μM Songorine.

Following a 24-hour treatment, RNA was obtained from the cellular samples using the previously mentioned method. RNA-seq samples were prepared using the NEBNext UltraTM RNA Library Prep Kit for the Illumina system, following the protocol provided by the manufacturer. At the OmicShare Bioinformatics Institute (Guangzhou, China), a HiSeq 3000 sequencer was utilized to conduct paired-end sequencing with a read length of 150 bp. HISAT2 (v2.0.5) was used to map the acquired RNA-seq reads to the Rnor_6.0 reference genome, employing the default settings. To evaluate the expression levels of various transcripts in the experimental groups, the calculation of Fragments per kilobase of exons per million mapped reads (FPKM) was performed (23). Genes that were significantly changed in response to LPS treatment, with or without Songorine treatment, were identified through differential expression analysis.

For data analysis and visualization, heatmaps of gene expression were generated to illustrate the overall gene expression patterns across different treatment groups. Furthermore, Kyoto Encyclopedia of Genes and Genomes (KEGG) pathway analyses were performed to gain insights into the biological functions and pathways affected by the treatments. Gene Set Enrichment Analysis (GSEA) was conducted using the gene ontology (GO) and KEGG databases, considering gene sets with absolute values of the Normalized Enrichment Score (|NES|) > 1, nominal p-val < 0.05, and False Discovery Rate (FDR) q-val < 0.25 as statistically significant. The top 15 pathways were selected based on the absolute values of NES for display. This analysis aimed to explore the shared metabolic pathways affected by Songorine treatment in LPS-treated RAW264.7 cells.

### 2.8 Rat OA model and treatments *in vivo*

Male Sprague-Dawley rats, weighing between 250 and 300 grams, were acquired from the Experimental Animal Center at Guangxi Medical University. The research involving animal trials was carried out following the Guidelines for Animal Experimentation of Guangxi Medical University, and the experimental protocol was approved by the Animal Ethics Committee of the institution (Approval No. 202201004). An anterior drawer test confirmed the success of the anterior cruciate ligament transection (ACLT) model in inducing OA in the rat’s knee joint, following a previously published method. The control group consisted of sham-operated rats (24).

After a month from the surgery, the ACLT rats were divided into three groups (n=6) randomly, and each group received an intra-articular injection of one of the following formulations: 1) saline, 2) 10 μM Songorine, or 3) 50 μM Songorine. Sham-operated rats served as a healthy control group. Injections were administered weekly. After the treatment commenced, the rats were euthanized at 4 and 8 weeks, and their knee joints were gathered for additional examination. Before micro-CT imaging, the knee joints were immersed in 4% paraformaldehyde for 24 hours to ensure fixation (25). A micro-computed tomography system (Micro-CT) (Quantum GX2, PerkinElmer) was utilized to conduct the imaging. The system operated at a resolution of 72 μm, with a 90 kV source and 88 μA current. Afterwards, the reconstructed datasets were analyzed using 3D analysis in the Mimics Research software (version 21.0) to assess osteophyte development. The volume of osteophytes or bone spurs was quantified to assess the extent of joint damage in knee osteoarthritis. Furthermore, each sample was given a macroscopic rating using a previously documented technique that quantitatively assesses the severity of OA (26).

### 2.9 Histology and immunohistochemistry

Following the imaging process, the knee joints underwent decalcification in a 10% (w/v) EDTA solution (pH 7.2) for a duration of four weeks prior to being enclosed in paraffin. For morphological analysis, 4 μm thick serial sagittal sections were prepared and stained with Safranin O/Fast Green. Additionally, some sections were stored for IHC analysis. The medial compartment of the knee was specifically analyzed at a distance of 50 μm between each level, with three levels of each section being examined for each sample. The OARSI scoring system was used to evaluate the extent of the osteoarthritis-like characteristics, which involved assessing three-level sections encompassing the femoral condyle and tibial plateau. Two impartial observers, who were unaware of the experimental groups, conducted this evaluation. Routine deparaffinization was performed on synovial and cartilage sections, followed by staining with hematoxylin and eosin (H&E). To assess the morphology of cartilage, sections of cartilage were treated to remove paraffin and then stained with a modified version of Safranin O and fast green.

To perform IHC analysis, the sections underwent deparaffinization and were then subjected to treatment with 3% H_2_O_2_ at ambient temperature for a duration of 15 minutes in order to remove any inherent peroxidase activity. Afterwards, the sections were subjected to antigen retrieval by incubating them with 0.25% EDTA trypsin (Procell, China, Cat. PB180225) at a temperature of 37°C for a duration of 20 minutes. Afterwards, the sections were obstructed using goat serum containing 10% concentration at a temperature of 37°C for a duration of 1 hour. This was succeeded by an overnight incubation with the primary antibody at a temperature of 4°C. Secondary antibody incubation and DAB color rendering were performed using an IHC kit. The microscope (VS120, OLYMPUS) was used to capture the resulting images, and the Image J software was utilized to quantify the percentage of positive areas.

### 2.10 Statistical analyses

The statistical analysis was performed using IBM SPSS Statistics software, specifically version 23.0. The data is presented as the average plus or minus the standard deviation (SD). Statistical analysis was conducted using either Student’s t-test or one-way ANOVA, with a p-value<0.05 indicating statistical significance. To guarantee the dependability of the findings, the experiments were conducted a minimum of three times (27, 28).

## 3 Results

### 3.1 Songorine redirecting macrophage polarization toward anti-inflammation

**Figure 1:**
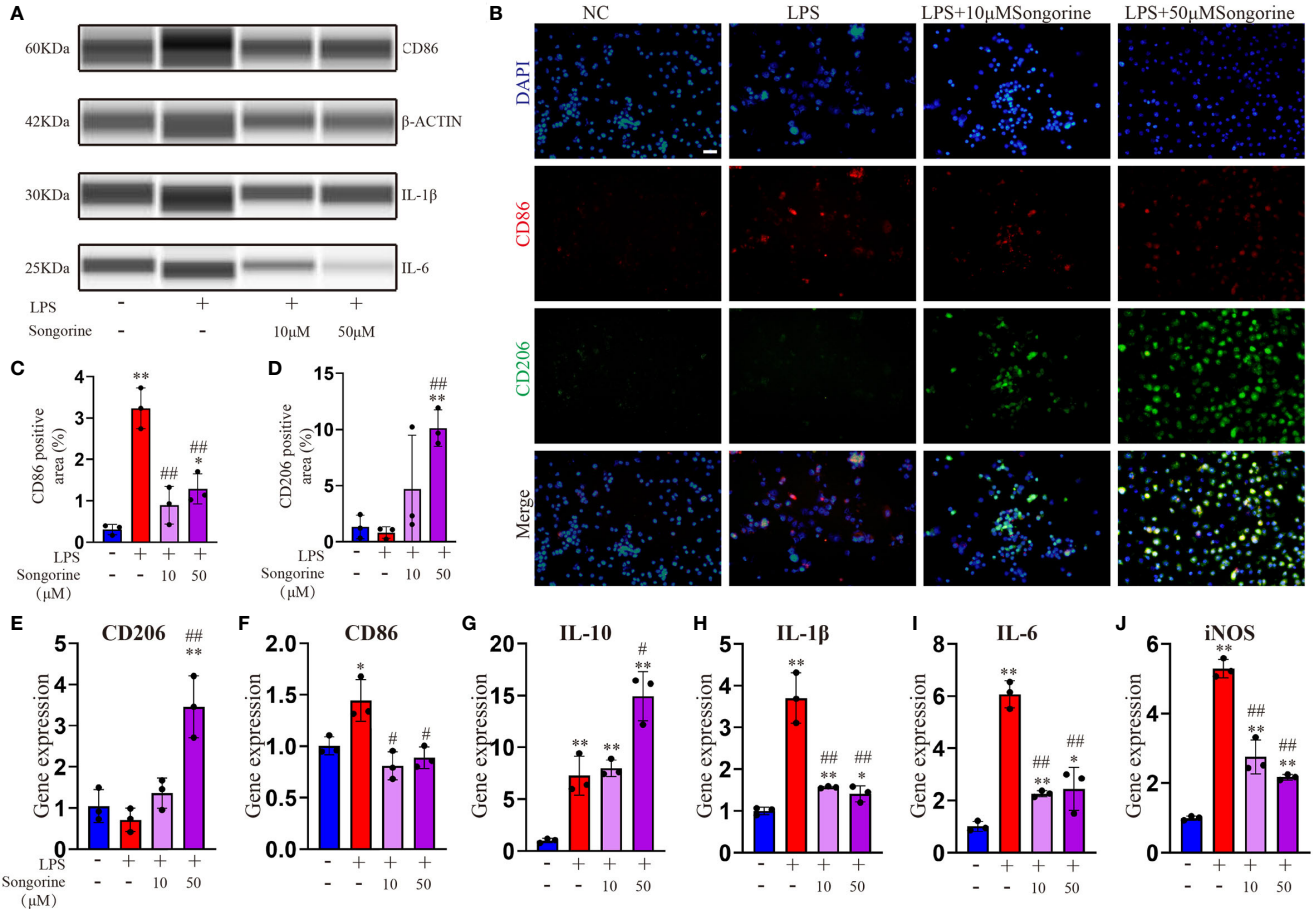
Effects of Songorine on macrophage polarization and inflammatory markers. Macrophages were treated as follows: untreated control (NC), LPS stimulation (LPS), and LPS stimulation with different concentrations of Songorine (LPS + 10 μM Songorine and LPS + 50 μM Songorine). **(A)** Protein expression of CD86, IL-1β, and IL-6 in macrophages after different treatments. **(B)** Representative images of double immunostaining of CD86 (M1 marker) and CD206 (M2 marker), Scale bar: 50 μm. **(C)** Quantification of CD86-positive cells and **(D)** CD206-positive cells in macrophages with different treatments. Immunofluorescence of CD86 and immunohistochemistry of CD206 were performed in macrophages after different treatments. The mRNA expression of M2 marker polarization **(E)** CD206, **(F)** M1 marker polarization (CD86), and pro-inflammatory cytokines, including **(G)** IL-10, **(H)** IL-1β, **(I)** IL-6, and **(J)** iNOS were analyzed. Statistical significance: * *p* < 0.05, ** *p* < 0.01 compared with normal control; # *p* < 0.05, ## *p* < 0.01 compared with the 1mg/mL LPS group.

In this section, we sought to comprehensively assess of Songorine’s effect on macrophages using various techniques. We noted a significant decrease in CD86-positive cells (M1 macrophages) (**Figures 1A–C**) and an increase in CD206-positive cells (M2 macrophages) following Songorine treatment (**Figures 1B, D**). Notably, Songorine not only affected M1 macrophages but also influenced M2 macrophage polarization. Stimulation with Songorine led to an upregulation in the expression of the M2 marker CD206 and the anti-inflammatory cytokine IL-10 (**Figures 1E–G**), indicative of a shift towards the anti-inflammatory M2 phenotype. We observed Songorine’s potent ability to modulate inflammatory responses. Specifically, Songorine exhibited a robust inhibition of M1 macrophage repolarization, resulting in a significant reduction in the expression of inflammatory cytokines IL-1β and IL-6 (**Figures 1A, H, I**). This underscores its efficacy in suppressing the induction of pro-inflammatory factors. Our exploration of inflammation-associated genes in RAW 264.7 cells, including IL-1β, IL-6, and iNOS, further validated the anti-inflammatory effects of Songorine (**Figures 1H–J**).

In summary, these findings collectively indicate that Songorine effectively modulates macrophage polarization, tipping the balance from the pro-inflammatory M1 phenotype towards the anti-inflammatory M2 phenotype. This remarkable ability holds promising therapeutic implications for managing inflammation in various diseases, including OA. The results not only provide compelling evidence for the therapeutic potential of Songorine in regulating macrophage polarization but also pave the way for further research and potential clinical applications as a targeted therapeutic agent for inflammatory disorders.

### 3.2 Songorine regulates metabolic shifts in macrophages during osteoarthritis

**Figure 2:**
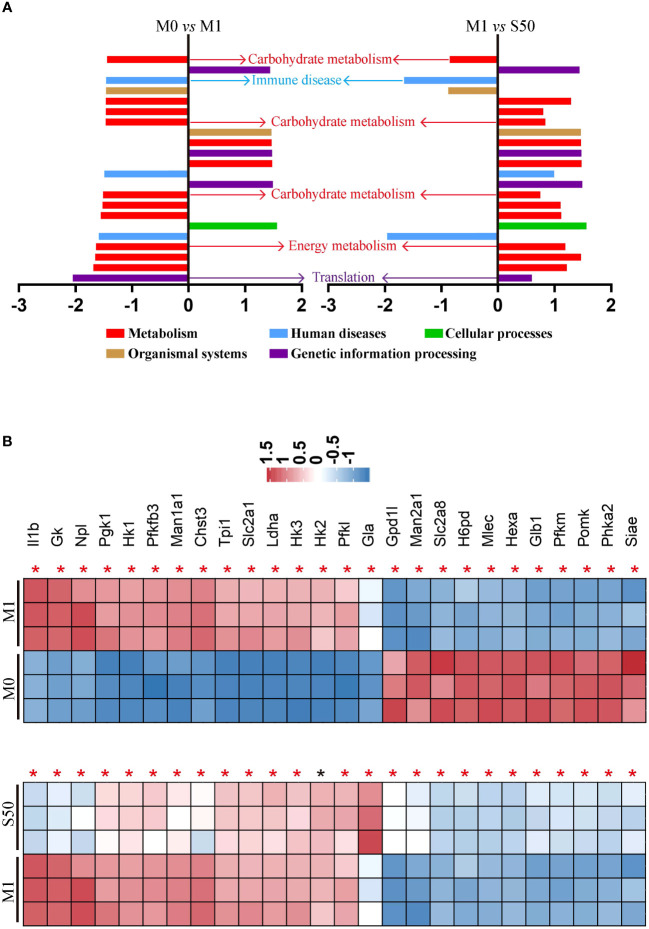
Songorine exhibits polarization effects of macrophages by regulating metabolic pathways. RNA-sequencing of chondrocytes with different groups. **(A)** GSEA analysis indicating common core biological pathways either upregulated or downregulated between data set 1 (M0 vs M1) and data set 2 (M1 vs S50). NES: normalized enrichment score. **(B)** Relative mRNA expression heat map of gene in glycolysis based on Log2FC in the two data sets (M0 compare with M1; M1 compare with S50). Asterisk indicates p<0.05 (black), p<0.01 (red).

To gain deeper insights into Songorine’s therapeutic properties in OA and uncover its molecular mechanisms, we conducted RNA-seq analysis. The results revealed significant variations in six biological process groups, as per the Kyoto Encyclopedia of Genes and Genomes (KEGG) database (**Supplementary Figure S1**). Analyzing data sets comparing M0 vs M1 and M1 vs S50 macrophages highlighted substantial alterations in lipid, amino acid, carbohydrate, and other metabolic pathways, with a total of 399 genes related to metabolism exhibiting changes. Notably, 103 genes within the carbohydrate metabolism showed significant expression changes when macrophages were stimulated with LPS. Gene set enrichment analysis (GSEA) showed that LPS activated pathways associated with metabolism, human diseases, organismal systems, and environmental information processing. However, these pathways were predominantly inhibited by Songorine in the S50 group, with carbohydrate metabolism in the metabolic pathway exhibiting the most significant changes (**Figure 2A**, **Supplementary Figure S2**). Importantly, Songorine’s inhibitory effects on LPS-induced glycolysis pathways and key genes were evident, normalizing the expression of glycolysis-related genes in inflammatory macrophages (**Figure 2B**). Our findings suggest a comprehensive regulatory role of Songorine in altering the expression of genes associated with various metabolic pathways. Specifically, the downregulation of carbohydrate metabolism genes points towards a metabolic reprogramming induced by Songorine, aligning with the observed shift from glycolysis to mitochondrial oxidative phosphorylation. This intricate interplay between gene expression and metabolic shifts highlights Songorine’s potential as a modulator of macrophage metabolism, contributing to its anti-inflammatory effects in the context of OA.

### 3.3 Songorine induces metabolic reprogramming in macrophages

**Figure 3:**
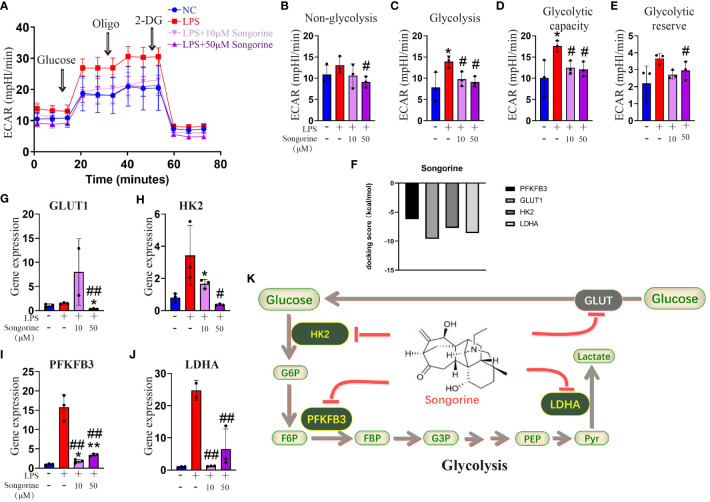
Songorine inhibits glycolysis by targets glycolytic enzymes. **(A)** Seahorse assay was performed to measure ECAR in different groups of macrophages. The ECAR was assessed by the Seahorse assay before and after sequential addition of Glucose, Oligomycin (Oligo), and 2-DG. **(B–E)** Non-glycolysis, glycolysis, glycolysis capacity and glycolytic reserve were calculated. **(F–I)** mRNA expression levels of GLUT1, HK2, PFKFB3 and LDHA in cells cultured with different treatments. Bar graphs and plots represent or include mean ± SD, respectively. **(J)** Docking scores of Songorine with PFKFB3, GLUT1, HK2, and LDHA. **(K)** Schematic illustration depicting the glycolytic regulation mechanism of Songorine. *p<0.05, **p<0.01 compared with the normal control; #p<0.05, ##p<0.01 compared with the 10 μg/mL IL-1β group.

To evaluate the impact of Songorine on macrophage metabolism during LPS stimulation, we monitored the ECAR. LPS-stimulated chondrocytes exhibited a significant increase in glycolysis compared to normal macrophages (**Figure 3A**). Inflammatory conditions enhanced all glycolysis-related parameters, demonstrating statistically significant differences (**Figures 3B–E**). However, Songorine suppressed glycolysis in inflamed macrophages, with glycolysis and glycolytic capacity decreasing in a concentration-dependent manner (**Figures 3C, D**), while non-glycolysis and glycolytic reserve were unaffected by LPS and Songorine (**Figures 3B, E**). To investigate the interaction between Songorine and metabolic pathways, molecular docking simulations were conducted. The molecular docking results, as shown in concentration-dependent, revealed that Songorine could tightly bind to key metabolic targets, including PFKFB3, GULT1, HK2, LDHA, PDH, and SUS. PFKFB3, GULT1, HK2, and LDHA are crucial enzymes in the glycolysis pathway, while PDH and SUS are key regulators of the tricarboxylic acid cycle. Songorine’s ability to bind tightly to key enzymes, such as PFKFB3, GULT1, HK2, and LDHA associated with glycolysis indicated a favorable binding of Songorine to glycolysis, forming strong hydrogen bonds with amino acid active groups and exhibiting strong binding energy (**Figure 3F**; **Supplementary Figure S3**). To further confirm Songorine’s direct inhibition of the glycolytic pathway through PFKFB3, GULT1, HK2, and LDHA, PCR assays showed that LPS stimulation promoted the expression of key glycolytic genes, namely PFKFB3, HK2, and LDHA, while Songorine significantly reversed the stimulatory effect of LPS, reducing the expression of these genes (**Figures 3G–J**). These results suggest that Songorine inhibits glycolysis by downregulating key metabolic bottleneck enzymes (as illustrated in **Figure 3K**), improving the metabolic microenvironment to inhibit M1 polarization, promote M2 polarization, and facilitate self-repair.

**Figure 4:**
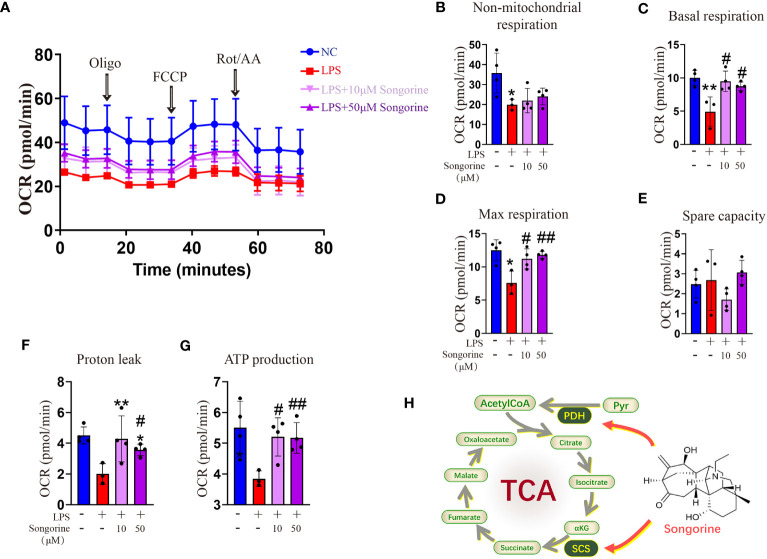
Songorine reshapes glucose metabolism in LPS-stimulated macrophages. **(A–G)** Seahorse assay was conducted to measure OCR in different macrophages groups. OCR was assessed before and after the sequential addition of Oligo, FCCP, and Rotenone/Antimycin (Rot/AA). **(H)** The schematic illustrates the mechanism through which Songorine transforms glucose metabolism, promoting TCA metabolism. Bar graphs and plots represent mean ± SD, respectively. *p < 0.05, **p < 0.01 compared with the normal control; #p < 0.05, ##p < 0.01 compared with the 10μg/mL IL-1β group.

Analysis of OCR revealed that IL-1β-treated cells exhibited inhibited mitochondrial oxidative phosphorylation (OXPHOS) (**Figure 4A**), including max respiration. Remarkably, Songorine treatment significantly enhanced OXPHOS and glucose metabolism, evidenced by increased basal respiration, max respiration, ATP production, and proton leak, even reaching normal levels (**Figures 4B–G**). These findings suggest that Songorine alters the metabolic status of LPS-treated macrophages, inhibiting glycolysis while preserving aerobic phosphorylation integrity (**Figure 4H**), thereby activating M2 polarization and suppressing inflammation.

### 3.4 Songorine suppresses macrophage oxidative stress through metabolic reprogramming

**Figure 5:**
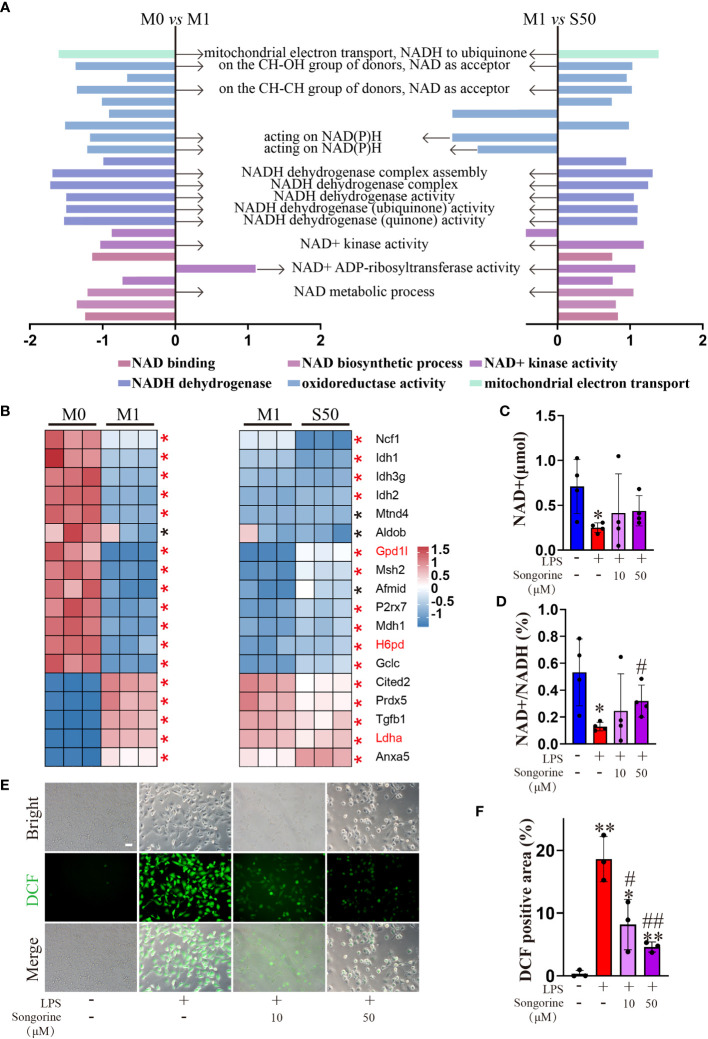
Songorine ameliorates oxidative stress in LPS-stimulated macrophages. **(A)** GSEA analysis reveals common core oxidative stress pathways either upregulated or downregulated between data set 1 (M0 vs M1) and data set 2 (M1 vs S50). NES, normalized enrichment score. **(B)** Heat map of relative mRNA expression for genes involved in oxidative stress based on Log2FC in the two datasets (M0 compared to M1; M1 compared to S50). Asterisks indicate p < 0.05 (black), p < 0.01 (red). **(C)** NAD+ content. **(D)** Ratio of NAD+/NADH. **(E, F)** Macrophages were cultured with different treatments, and ROS levels were assessed using DCFH-DA as a probe, Scale bar: 50 μm. Bar graphs and plots represent mean ± SD, respectively. *p < 0.05, **p < 0.01 compared with the normal control; #p < 0.05, ##p < 0.01 compared with the 10μg/mL IL-1β group.

Interestingly, Songorine exhibits a concentration-dependent increase in proton leak (**Figure 4F**), a phenomenon known to mitigate ROS production and consequently inhibit the onset of oxidative stress. GSEA results reveal that LPS-stimulated macrophages are enriched in the oxidative stress pathway, promoting the occurrence of oxidative stress (**Figure 5A**). However, the addition of Songorine to LPS-stimulated macrophages significantly reverses the occurrence of oxidative stress, with the most notable changes observed in pathways related to NAD metabolism, such as NADH dehydrogenase complex assembly, NADH dehydrogenase complex, NADH dehydrogenase (ubiquinone) activity, NAD(+) activity, and NAD metabolic process. Gene enrichment analysis indicates that NAD-related gene expression is significantly inhibited in LPS-stimulated macrophages, while genes associated with oxidative stress show a marked increase (**Figure 5B**). In contrast, Songorine inhibits oxidative stress and promotes the regulation of NAD-related gene expression. Notably, Gpd1l, H6pd, and Ldha, which are significantly enriched in metabolism and oxidative stress, emphasize that these genes not only play a regulatory role in metabolism but also hold importance in oxidative stress or NAD synthesis. Songorine, as previously demonstrated to target and inhibit LDHA expression, not only regulates metabolism but also suppresses the occurrence of oxidative stress. Determining NAD content is crucial in our study as NAD plays a pivotal role in cellular metabolism and redox reactions. Measurement of NADH and NAD levels in macrophages aligns with gene expression results, confirming that LPS stimulation inhibits NAD+ production (**Figure 5C**) and NAD(+)/NADH ratio (**Figure 5D**), whereas Songorine counteracts this effect in a concentration-dependent manner, exhibiting optimal results at 50μM, approaching normal levels. Furthermore, it is revealed that Songorine decreases the production of ROS in LPS-stimulated M1 macrophages, as indicated by reduced green fluorescence (**Figures 5E, F**).

### 3.5 Songorine modulates the inflammatory microenvironment to facilitate chondrocyte repair

**Figure 6:**
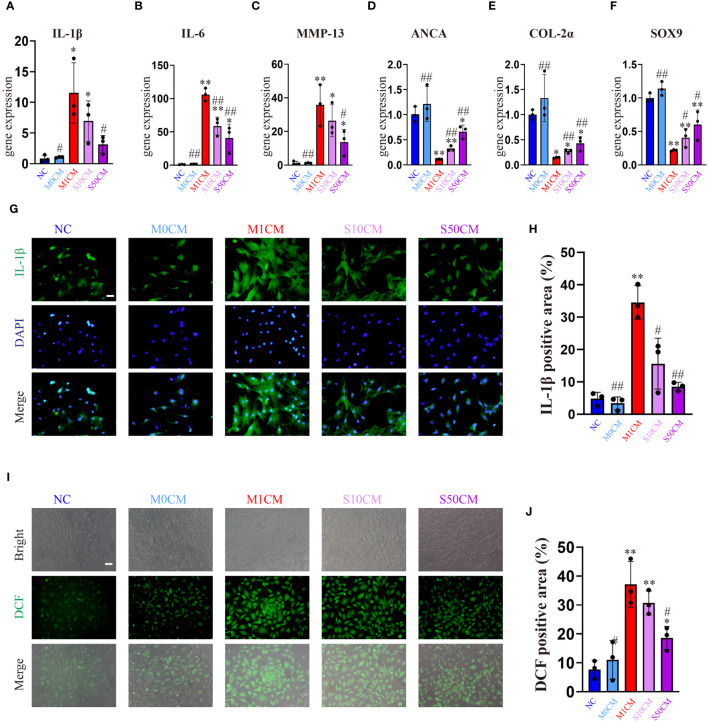
Songorine exerts a dual effect by protecting chondrocytes and remodeling the macrophage-induced inflammatory microenvironment. Conditioned media from M1 macrophages, 10μM Songorine-treated M1 macrophages, and 50 μM Songorine-treated M1 macrophages were labeled as M0CM, M1CM, 10SCM, and 50SCM, respectively. **(A–F)** Evaluation of mRNA expression levels of anabolic and pro-inflammatory cytokines, including IL-1β, IL-6, MMP-13, ACAN, COL-2, and SOX-9. **(G, H)** Immunofluorescence and quantification of IL-1β in chondrocytes after exposure to different treatments using macrophage-conditioned media. Scale bar: 50 μm. **(I, J)** Chondrocytes were cultured with different conditioned medium, and ROS levels were measured using DCFH-DA as a probe, Scale bar: 50 μm. ^*^ *p*<0.05, ^**^ *p*<0.01 compared with normal control; ^#^ *p*<0.05, ^##^ *p*<0.01 compared with the M1CM group.

Control and M0CM groups exhibit low expression of inflammatory genes, while the M1CM group shows elevated levels of *IL-1β*, *IL-6* and *MMP13* (**Figures 6A–C**) alongside decreased anabolic genes *ACAN*, *COL-2α*, and *SOX9* (**Figures 6D–F**). This indicates chondrocyte stimulation within an inflammatory microenvironment. Treatment with S10CM and S50CM significantly reduces the expression of inflammatory and catabolic genes compared to M1CM, upregulating anabolic genes. Songorine effectively shifts the immune microenvironment from inflammatory to anti-inflammatory states. As expected, IF analysis shows a substantial decrease in IL-1β gene expression with Songorine treatment at different concentrations compared to the M1CM group (**Figures 6G, H**). Monitoring ROS content using DCFH-DA reveals that Songorine concentration-dependently decreases ROS content, highlighting its potent ROS-scavenging capacity (**Figures 6I, J**).

These findings demonstrate that Songorine suppresses the inflammatory microenvironment enriched with pro-inflammatory factors and promotes anabolic processes in chondrocytes. Songorine’s immunomodulatory effects on macrophage reprogramming shift the inflammatory microenvironment toward anti-chondrogenic conditions, impacting cellular functions and contributing to the regulation of microenvironments in the joint capsule affected by OA.

### 3.6 Songorine protects against osteoarthritis in a surgically induced *in vivo* model

**Figure 7:**
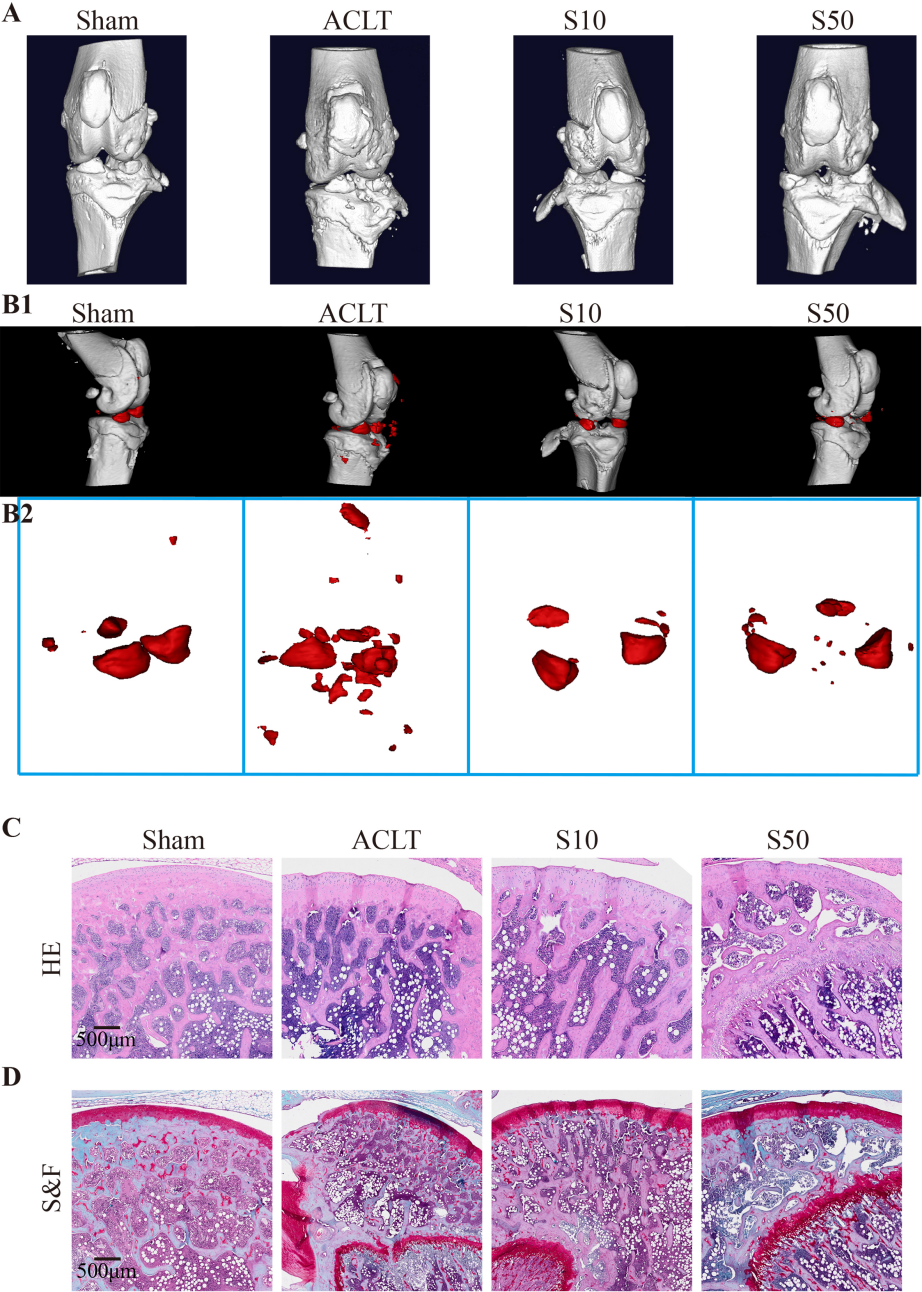
Songorine effectively mitigates the remodeling and cartilage damage in the knee joint following ACLT surgery. The sham-operated group is denoted as “sham,” the ACLT-operated group as “OA,” and the ACLT-operated groups treated with 10μM or 50μM Songorine as “S10” or “S50.” **(A)** Three-dimensional micro-CT images vividly portray pathological structural changes in rat knees. **(B1, B2)** Three-dimensional micro-CT images reveal the formation of calcified meniscus and synovial tissue among the groups, with the region marked in red indicating calcified tissue. **(C)** Representative images of H&E-stained sections from rats treated with or without Songorine for 4 weeks. Scale bar: 500 μm. **(D)** Representative images of Safranin O & Fast Green-stained sections from rats treated with or without Songorine for 4 weeks. Scale bar: 500 μm.

To assess the *in vivo* efficacy of Songorine in treating OA, we employed a rat model with ACLT as the OA induction method. ACLT rats were randomly divided into three groups receiving saline, 10μM Songorine, and 50μM Songorine treatments, respectively. Sham-operated rats served as healthy controls. Treatment initiation occurred on the 30th day post-surgery, repeated every five days until day 60. Rats were euthanized for joint collection, and Micro-CT imaging was performed. Joints were dissected, exposing synovium and cartilage, and photographed (**Supplementary Figure S4A**). In the ACLT group, synovium thickening and adhesion to cartilage indicated severe synovitis, with substantial damage to the cartilage surface. Songorine treatment preserved a smooth cartilage surface, comparable to the sham group, affirming its protective effect against ACLT-induced cartilage damage (**Supplementary Figure S4B**). The high concentration group exhibited more significant benefits against cartilage damage than the low concentration group. Micro-CT images (**Figures 7A, B1, B2**) displayed remarkable bone density reduction in the ACLT group, Songorine treatment effectively preserved the integrity of cartilage and bone structures, and suppressed the formation of osteophytes or bone spurs (**Supplementary Figure S4C**). Songorine exhibited the best outcome, maintaining the intact subchondral bone structure. S&F and HE staining (**Figures 7C, D**) illustrated proteoglycan loss and reduced articular cartilage thickness post-ACLT, whereas Songorine-treated ACLT rats showed significant inhibition of cartilage degeneration. OARSI scores further confirmed these protective results (**Supplementary Figure S4D**).

**Figure 8:**
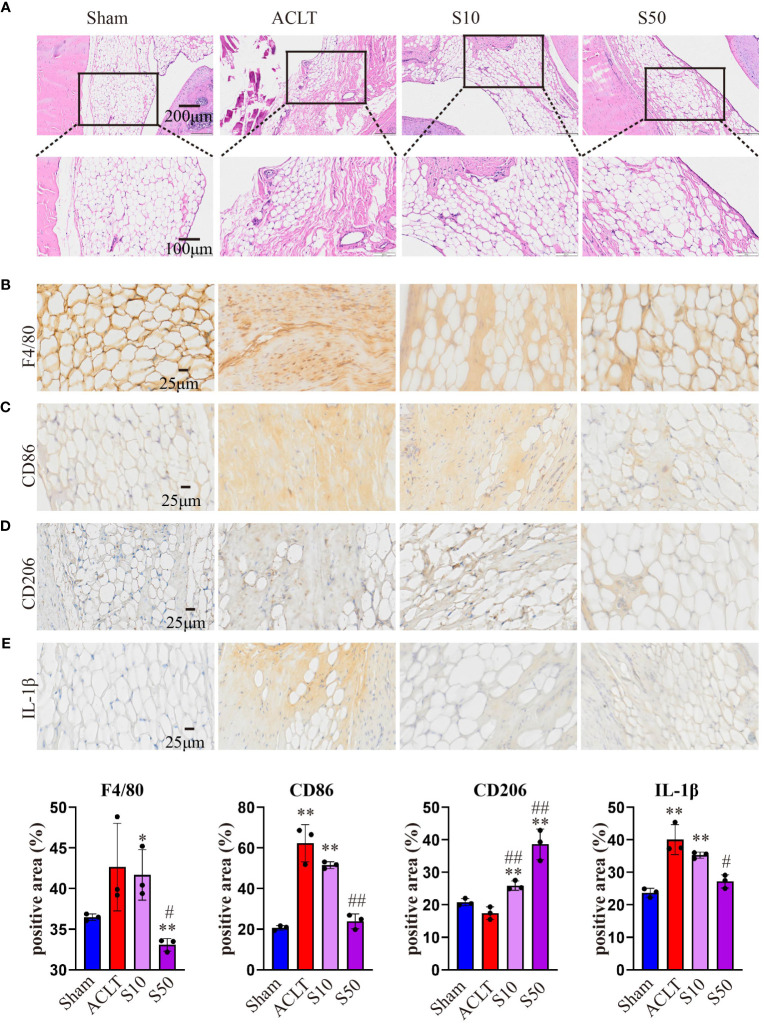
Staining evaluations of Songorine in delaying synovial membrane damage in ACLT-induced OA. **(A)** H&E-stained synovial membrane from rats treated with or without Songorine for 4 weeks. **(B–E)** Immunohistochemistry staining for F4/80, CD86, CD206, and IL-1β in the synovial membrane. Scale bar: 25μm.

Synovial inflammation crucially influences OA progression; hence, we conducted a histological analysis of synovium. **Figure 8** shows that OA destroyed the synovial reticular structure, with inflammatory cell infiltration causing thickening and disruption of the normally porous structure. Songorine maintained the synovial reticular structure and suppressed inflammatory cell infiltration. To explore Songorine’s mechanisms in OA treatment, we used an immunohistochemical assay to evaluate synovial inflammation and infiltrated macrophage phenotypes. F4/80, CD86 (M1 biomarker), and CD206 (M2 biomarker) were employed for macrophage identification, and quantitative analysis was conducted. In ACLT synovium, F4/80-stained macrophages increased, however, Songorine demonstrates a concentration-dependent reduction in macrophage infiltration while preserving the normal structure of the synovial tissue (**Figure 8A**). CD86-positive area increased, while CD206-positive area remained similar to healthy synovium. Songorine treatment decreased CD86-positive area increased In ACLT synovium, while CD206-positive area remained similar to healthy synovium. treatment with Songorine led to a decrease in the CD86-positive area and an increase in the CD206-positive area (**Figures 8B, C**), indicating a reprogramming of infiltrated M1 macrophages into the M2 phenotype. Additionally, Songorine increased M2-type macrophages (CD206-positive cells) in synovium and decreased IL-1β expression (**Figure 8E**). Songorine’s anti-inflammatory effects were also evident in cartilage tissue, with increased IL-1β expression in the ACLT group (**Supplementary Figure S5A**) reduced by Songorine. Simultaneously, Songorine promoted SOX9 expression (**Supplementary Figure S5B**), vital for cartilage maintenance.

## 4 Discussion

A key finding of our study is the role of metabolic pathways in Songorine’s anti-inflammatory mechanism. Metabolic pathways play a crucial role in regulating cellular functions, and their dysregulation is increasingly recognized as a key contributor to inflammatory processes in OA. RNA-seq analysis revealed significant variations in various metabolic pathways, particularly those related to carbohydrate metabolism. Songorine effectively reversed LPS-induced glycolysis pathways, indicating its potential to influence macrophage polarization through metabolic alterations. Molecular docking simulations further supported Songorine’s binding affinity to key glycolysis enzymes, providing insights into its direct inhibition of the glycolytic pathway.

OA is a prevalent degenerative joint disorder characterized by inflammation, cartilage degradation, and structural changes (29). Recent research has delved into the molecular mechanisms underlying OA and explored novel therapeutic agents. Macrophages play a crucial role in the pathogenesis of OA, contributing to the inflammatory milieu within the joint (30). Our results demonstrate that Songorine significantly influences the inflammatory microenvironment within the joint. Through a comprehensive analysis using PCR, Western blot, and immunofluorescence techniques, we observed a notable elevation in pro-inflammatory cytokines, indicating a crucial role in immune function regulation. Importantly, Songorine exhibited a remarkable capacity to modulate macrophage polarization, shifting the balance from the pro-inflammatory M1 phenotype to the anti-inflammatory M2 phenotype. This effect was evidenced by a reduction in M1 macrophage markers and an increase in M2 macrophage markers.

Our study unveiled Songorine’s ability to induce metabolic reprogramming in macrophages during LPS stimulation. LPS stimulation induces a robust increase in glycolytic activity in macrophages, facilitating rapid ATP production and providing essential intermediates for biosynthetic pathways. Key enzymes in glycolysis, such as hexokinase and pyruvate kinase, are upregulated to meet the heightened energy demands of activated macrophages (31). This metabolic shift towards glycolysis not only sustains the energy requirements for immune responses but also contributes to the production of inflammatory cytokines. Notably, Songorine suppressed glycolysis in inflamed macrophages in a concentration-dependent manner. The interaction between Songorine and metabolic pathways, as indicated by molecular docking simulations, highlighted its tight binding to crucial enzymes involved in glycolysis and the tricarboxylic acid cycle. Metabolic rewiring towards glycolysis emerges as a hallmark of M1 macrophages, characterized by heightened glucose uptake and lactate production. Glycolytic enzymes, such as hexokinase and pyruvate kinase, are upregulated, fueling the energetic demands of M1 polarization (32, 33). Conversely, M2 macrophages exhibit a preference for oxidative phosphorylation, relying on mitochondrial metabolism for energy production. Enhanced fatty acid oxidation (FAO) and tricarboxylic acid (TCA) cycle activity characterize the metabolic landscape of M2 polarization (34). In our study, Songorine reversed glycolysis pathways, as evidenced by specific changes in gene expression and metabolic profiles, suggests a metabolic reprogramming associated with anti-inflammatory effects. This reversal is likely influences macrophage phenotype, reduces the production of inflammatory mediators, and contributes to the resolution of inflammation. The downstream consequences encompass a shift towards tissue repair, maintenance of cellular homeostasis, and mitigation of oxidative stress, collectively contributing to the potential therapeutic benefits of Songorine in the context of osteoarthritis. This metabolic reprogramming facilitated the inhibition of M1 polarization, promotion of M2 polarization, and support for self-repair processes, including ECM remodeling, enhanced chondrocyte activity, anti-inflammatory signaling, maintenance of redox balance, and modulation of immune responses. The observed metabolic changes collectively create a microenvironment that encourages the innate repair mechanisms of the joint, thereby contributing to the recovery from OA.

Oxidative damage to proteins, lipids, and DNA exacerbates the breakdown of cartilage tissues, a hallmark of OA progression (35). Macrophages in the OA synovium exhibit a skewed polarization profile, with an overabundance of pro-inflammatory M1 macrophages. These M1 macrophages are potent sources of ROS and pro-inflammatory cytokines, creating a microenvironment conducive to oxidative stress-induced damage (35, 36). An intriguing aspect of Songorine’s mechanism is its impact on macrophage oxidative stress. By enhancing proton leak, Songorine significantly reduced oxidative stress in LPS-stimulated macrophages. Proton leakage serves as a critical mechanism to regulate mitochondrial membrane potential (ΔΨm) and, consequently, ROS generation. Excessive ROS, implicated in oxidative stress, contribute to cellular damage and various pathological conditions. Proton leakage acts as a regulatory brake, preventing ROS accumulation and averting oxidative stress-induced injury to cellular components (37). Gene enrichment analysis revealed the inhibition of oxidative stress-related pathways, emphasizing the importance of NAD metabolism. NAD’s involvement in both glycolysis and oxidative phosphorylation positions it as a central player in energy metabolism. This dual engagement plays a critical role in cellular respiration and influences the production of ROS (38). NAD levels can influence proton leakage in mitochondria, thereby impacting the generation of ROS. A dynamic equilibrium exists, wherein adequate NAD levels can modulate proton flux, contributing to the prevention of excessive ROS production (39, 40). Songorine’s ability to regulate NAD-related gene expression and maintain intracellular ROS balance suggests its potential as an antioxidant in the context of inflammatory disorders.

Our findings extend beyond macrophage modulation, demonstrating Songorine’s ability to shift the immune microenvironment from inflammatory to anti-inflammatory states. Preliminary findings provided insights into similar approaches where the secretome of immune cells has been implicated in influencing the behavior of neighboring cells, particularly in the context of joint disorders (41, 42). In chondrocytes, Songorine suppressed the expression of inflammatory and catabolic genes while promoting anabolic processes. This immunomodulatory effect contributes to the regulation of microenvironments in the joint capsule affected by OA, highlighting the potential of using conditioned media as a valuable tool to understand the broader impact of Songorine on the joint microenvironment and paving the way for novel therapeutic strategies.

The translational significance of our study was underscored by *in vivo* experiments employing a rat model of surgically induced OA. Songorine treatment, especially at a higher concentration, demonstrated protective effects against cartilage damage, synovial inflammation, and bone density reduction. Histological analyses further confirmed Songorine’s ability to maintain synovial structure, reprogram macrophages, and attenuate inflammatory responses in articular cartilage. In the upcoming research phase, our primary focus will be on enhancing the *in vivo* anti-inflammatory and cartilage-protective effects of Songorine. This involves exploring innovative delivery systems such as hydrogel carriers to facilitate more efficient and sustained release of Songorine within the joint cavity. Additionally, we aim to delve into more molecular targets of Songorine in osteoarthritis, paving the way for the development of new targeted drugs for the treatment of this condition.

In conclusion, Songorine demonstrates a protective effect on cartilage and synovium, effectively transforming macrophages into an anti-inflammatory M2 phenotype while inhibiting glycolysis and enhancing oxidative phosphorylation. This metabolic reprogramming is associated with a reduction in oxidative stress in macrophages. These findings provide a solid foundation for the potential use of Songorine as a therapeutic agent for osteoarthritis, encouraging further research into its specific molecular targets and clinical applications.

## Data availability statement

The datasets presented in this study can be found in online repositories. The names of the repository/repositories and accession number(s) can be found below: PRJNA1069788 (SRA).

## Ethics statement

The animal studies were approved by Experimental Animal Center at Guangxi Medical University. The studies were conducted in accordance with the local legislation and institutional requirements. Written informed consent was obtained from the owners for the participation of their animals in this study.

## Author contributions

X-XH: Conceptualization, Formal analysis, Investigation, Methodology, Software, Visualization, Writing – original draft. Y-JH: Formal analysis, Investigation, Software, Writing – original draft. C-LH: Software, Writing – original draft. Q-QX: Software, Resources, Supervision, Validation, Visualization, Writing – review & editing. Q-JW: Supervision, Validation, Writing – review & editing, Data curation, Funding acquisition, Project administration.

## Supplementary material

Supplementary Figure 1 (KEGG) database classified 44 pathways in data set 1(M0 vs M1) and 40 pathways in data set 2(M1 vs S50) into six biological process groups.

Supplementary Figure 2 In the GSEA analysis comparing group M1 versus group S50, Songorine was found to positively regulate glucose metabolism, correcting the metabolic dysregulation induced by LPS in macrophages.

Supplementary Figure 3 The molecular docking diagram of Songorine binding with six metabolic targets. molecular model of Songorine (left); Three-dimensional (3D) binding model (right) and complexes (down) with the important interacting residues depicted in bright color. The backbone of the protein was rendered in tube and appears blue; Songorine is rendered silver gray; the yellow dash represents the hydrogen bond distance.

Supplementary Figure 4
*In vivo* therapeutic effect of Songorine in ACLT-Induced OA rats. **(A)** The gross observations of knee joint cartilage (femur and tibial plateau), the sham-operated group is marked as sham, ACLT-operated group are marked as OA, and ACLT-operated group treated with 10μM or 50μM Songorine are marked as S10 or S50. **(B)** The Pelletier score of knee joint cartilage. **(C)** Quantification of the volume of calcified meniscus and synovial tissue (Cal Tis.V). **(D)** Analysis of OA-like phenotype severity after ACLT surgery using the Osteoarthritis Research Society International (OARSI) score system. **p*<0.05, ***p*<0.01, versus sham group; #*p*<0.05, ##*p*<0.01 compared with the ACLT group.

Supplementary Figure 5 Immunohistochemistry staining for IL-1β and SOX9 in knee joint medial compartment cartilage. Scale bar: 25μm.

## References

[1] Fan X Wu X Trevisan Franca De Lima L Stehbens S Punyadeera C Webb R. The deterioration of calcified cartilage integrity reflects the severity of osteoarthritis-A structural, molecular, and biochemical analysis. FASEB J Off Publ Fed Am Societies Exp Biol (2022) 36(2):e22142. doi: 10.1096/fj.202101449R 35032407

[2] Khury F Oltmanns M Unseld T Fuchs M Reichel H Faschingbauer M. Which knee phenotypes exhibit the strongest correlation with cartilage degeneration? Clin orthopaedics related Res (2023). doi: 10.1097/CORR.0000000000002831 PMC10871747 37703204

[3] Tuckermann J Adams R. The endothelium-bone axis in development, homeostasis and bone and joint disease. Nat Rev Rheumatol (2021) 17(10):608–20. doi: 10.1038/s41584-021-00682-3 PMC7612477 34480164

[4] Wang Q Lepus C Raghu H Reber L Tsai M Wong H. IgE-mediated mast cell activation promotes inflammation and cartilage destruction in osteoarthritis. eLife (2019) 8:e39905. doi: 10.7554/eLife.39905 31084709 PMC6516833

[5] Courties A Olmer M Myers K Ordoukhanian P Head S Natarajan P. Human-specific duplicate CHRFAM7A gene is associated with more severe osteoarthritis and amplifies pain behaviours. Ann rheumatic diseases. (2023) 82(5):710–8. doi: 10.1136/ard-2022-223470 PMC10101906 36627169

[6] Shkhyan R Flynn C Lamoure E Sarkar A Van Handel B Li J. Inhibition of a signaling modality within the gp130 receptor enhances tissue regeneration and mitigates osteoarthritis. Sci Trans Med (2023) 15(688):eabq2395. doi: 10.1126/scitranslmed.abq2395 PMC10792550 36947594

[7] Yang Y Jian Y Liu Y Xie Q Yu H Wang B. Heilaohuacid G, a new triterpenoid from Kadsura coccinea inhibits proliferation, induces apoptosis, and ameliorates inflammation in RA-FLS and RAW 264.7 cells via suppressing NF-. Phytotherapy Res PTR (2022) 36(10):3900–10. doi: 10.1002/ptr.7527 36104304

[8] Hsieh C Wang C Tayo L Deng S Tsai P Lee C. *In vitro* and in *vivo* anti-osteoarthritis effects of tradition Chinese prescription Ji-Ming-San. J Ethnopharmacol (2023) 305:116084. doi: 10.1016/j.jep.2022.116084 36584922

[9] Yao L Wu X Jiang X Shan M Zhang Z Li Y. Subcellular compartmentalization in the biosynthesis and engineering of plant natural products. Biotechnol advances. (2023) 69:108258. doi: 10.1016/j.biotechadv.2023.108258 37722606

[10] Lephatsi M Choene M Kappo A Madala N Tugizimana F. An integrated molecular networking and docking approach to characterize the metabolome of Helichrysum splendidum and its pharmaceutical potentials. Metabolites (2023) 13(10):1104. doi: 10.3390/metabo13101104 37887429 PMC10609414

[11] Han J Lee E Park W Ha K Chung H. Natural compounds as lactate dehydrogenase inhibitors: potential therapeutics for lactate dehydrogenase inhibitors-related diseases. Front Pharmacol (2023) 14:1275000. doi: 10.3389/fphar.2023.1275000 37915411 PMC10616500

[12] Zhang H Dong R Zhang P Wang Y. Songorine suppresses cell growth and metastasis in epithelial ovarian cancer via the Bcl−2/Bax and GSK3β/β−catenin signaling pathways. Oncol Rep (2019) 41(5):3069–79. doi: 10.3892/or.2019.7070 30896826

[13] Ren Z Zhang H Yang L Wang Z Xiong J Zheng P. Targeted preparation and recognition mechanism of broad-spectrum antibody specific to Aconitum alkaloids based on molecular modeling and its application in immunoassay. Analytica Chimica Acta (2022) 1222:340011. doi: 10.1016/j.aca.2022.340011 35934421

[14] He G Wang X Liu W Li Y Shao Y Liu W. Chemical constituents, pharmacological effects, toxicology, processing and compatibility of Fuzi (lateral root of Aconitum carmichaelii Debx): A review. J ethnopharmacology. (2023) 307:116160. doi: 10.1016/j.jep.2023.116160 36773791

[15] Yao J Chen C Sun Y Lin Y Tian Z Liu X. Higenamine exerts antidepressant effect by improving the astrocytic gap junctions and inflammatory response. J Affect Disord (2023) 348:107–15. doi: 10.1016/j.jad.2023.12.020 38101523

[16] Khan H Nabavi S Sureda A Mehterov N Gulei D Berindan-Neagoe I. Therapeutic potential of songorine, a diterpenoid alkaloid of the genus Aconitum. Eur J Medicinal Chem (2018) 153:29–33. doi: 10.1016/j.ejmech.2017.10.065 29133056

[17] Zhao X Wang Y Li Y Chen X Yang H Yue J. Songorine, a diterpenoid alkaloid of the genus Aconitum, is a novel GABA(A) receptor antagonist in rat brain. Neurosci Lett (2003) 337(1):33–6. doi: 10.1016/S0304-3940(02)01299-5 12524165

[18] Ameri A. Effects of the Aconitum alkaloid songorine on synaptic transmission and paired-pulse facilitation of CA1 pyramidal cells in rat hippocampal slices. Br J Pharmacol (1998) 125(3):461–8. doi: 10.1038/sj.bjp.0702100 PMC1565649 9806328

[19] Xiong J He J Zhu J Pan J Liao W Ye H. Lactylation-driven METTL3-mediated RNA mA modification promotes immunosuppression of tumor-infiltrating myeloid cells. Mol Cell (2022) 82(9):1660–77.e10. doi: 10.1016/j.molcel.2022.02.033 35320754

[20] Van Acker Z Perdok A Hellemans R North K Vorsters I Cappel C. Phospholipase D3 degrades mitochondrial DNA to regulate nucleotide signaling and APP metabolism. Nat Commun (2023) 14(1):2847. doi: 10.1038/s41467-023-38501-w 37225734 PMC10209153

[21] Reddy V Chinta K Saini V Glasgow J Hull T Traylor A. Mycobacterium tuberculosisFerritin H deficiency in myeloid compartments dysregulates host energy metabolism and increases susceptibility to infection. Front Immunol (2018) 9:860. doi: 10.3389/fimmu.2018.00860 29774023 PMC5943674

[22] Jia R Du J Cao L Feng W He Q Xu P. Application of transcriptome analysis to understand the adverse effects of hydrogen peroxide exposure on brain function in common carp (Cyprinus carpio). Environ Pollut (Barking Essex 1987). (2021) 286:117240. doi: 10.1016/j.envpol.2021.117240 33991737

[23] Lan C Chen C Qu S Cao N Luo H Yu C. Inhibition of DYRK1A, via histone modification, promotes cardiomyocyte cell cycle activation and cardiac repair after myocardial infarction. EBioMedicine (2022) 82:104139. doi: 10.1016/j.ebiom.2022.104139 35810562 PMC9278077

[24] Guo H Yin W Zou Z Zhang C Sun M Min L. Quercitrin alleviates cartilage extracellular matrix degradation and delays ACLT rat osteoarthritis development: An in *vivo* and in *vitro* study. J Adv Res (2021) 28:255–67. doi: 10.1016/j.jare.2020.06.020 PMC7753236 33364061

[25] Zhu J Zhu Y Xiao W Hu Y Li Y. Instability and excessive mechanical loading mediate subchondral bone changes to induce osteoarthritis. Ann Trans Med (2020) 8(6):350. doi: 10.21037/atm.2020.02.103 PMC7186756 32355794

[26] Kou L Huang H Tang Y Sun M Li Y Wu J. Opsonized nanoparticles target and regulate macrophage polarization for osteoarthritis therapy: A trapping strategy. J Control Release. (2022) 347:237–55. doi: 10.1016/j.jconrel.2022.04.037 35489544

[27] Lu E Wu L Chen B Xu S Fu Z Wu Y. Maternal serum tRNA-derived fragments (tRFs) as potential candidates for diagnosis of fetal congenital heart disease. J Cardiovasc Dev Dis (2023) 10(2):78. doi: 10.3390/jcdd10020078 36826574 PMC9968204

[28] Li Y Zang H Zhang X Huang G. Exosomal Circ-ZNF652 Promotes Cell Proliferation, Migration, Invasion and Glycolysis in Hepatocellular Carcinoma via miR-29a-3p/GUCD1 Axis. Cancer Manag Res (2020) 12:7739–51. doi: 10.2147/CMAR.S259424 PMC7473989 32943922

[29] Li X Tao H Zhou J Zhang L Shi Y Zhang C. MAGL inhibition relieves synovial inflammation and pain via regulating NOX4-Nrf2 redox balance in osteoarthritis. Free Radical Biol Med (2023) 208:13–25. doi: 10.1016/j.freeradbiomed.2023.07.019 37516370

[30] Liao Z Umar M Huang X Qin L Xiao G Chen Y. Transient receptor potential vanilloid 1: A potential therapeutic target for the treatment of osteoarthritis and rheumatoid arthritis. Cell Prolif (2023) e13569. doi: 10.1111/cpr.13569 37994506 PMC10905355

[31] Mi Y Tang M Wu Q Wang Y Liu Q Zhu P. NMAAP1 regulated macrophage polarizion into M1 type through glycolysis stimulated with BCG. Int immunopharmacology. (2023) 126:111257. doi: 10.1016/j.intimp.2023.111257 37988910

[32] Palmieri E Holewinski R McGinity C Pierri C Maio N Weiss J. Pyruvate dehydrogenase operates as an intramolecular nitroxyl generator during macrophage metabolic reprogramming. Nat Commun (2023) 14(1):5114. doi: 10.1038/s41467-023-40738-4 37607904 PMC10444860

[33] Gauthier T Yao C Dowdy T Jin W Lim Y Patiño L. TGF-β uncouples glycolysis and inflammation in macrophages and controls survival during sepsis. Sci Signaling (2023) 16(797):eade0385. doi: 10.1126/scisignal.ade0385 PMC11145950 37552767

[34] Peace C O'Carroll S O'Neill L. Fumarate hydratase as a metabolic regulator of immunity. Trends Cell Biol (2023) 6:S0962-8924(23)00209-X. doi: 10.1016/j.tcb.2023.10.005 37940417

[35] Lei L Cong R Ni Y Cui X Wang X Ren H. Dual-functional injectable hydrogel for osteoarthritis treatments. Adv Healthc Mater (2023) e2302551. doi: 10.1002/adhm.202302551 37988224

[36] Miao M Su Q Cui Y Bahnson E Li G Wang M. Redox-active endosomes mediate α5β1 integrin signaling and promote chondrocyte matrix metalloproteinase production in osteoarthritis. Sci Signaling (2023) 16(809):eadf8299. doi: 10.1126/scisignal.adf8299 PMC10666734 37906629

[37] Chen C Zhang L Jin Z Kasumov T Chen Y. Mitochondrial redox regulation and myocardial ischemia-reperfusion injury. Am J Physiol Cell Physiol (2022) 322(1):C12–23. doi: 10.1152/ajpcell.00131.2021 PMC8721908 34757853

[38] Esaki N Matsui T Tsuda T. viaLactate induces the development of beige adipocytes an increase in the level of reactive oxygen species. Food Funct (2023) 14(21):9725–33. doi: 10.1039/D3FO03287F 37817572

[39] Li Q Zhou M Chhajed S Yu F Chen S Zhang Y. N-hydroxypipecolic acid triggers systemic acquired resistance through extracellular NAD(P). Nat Commun (2023) 14(1):6848. doi: 10.1038/s41467-023-42629-0 37891163 PMC10611778

[40] Kang H Kim S Lee JY Kim B. Inhibitory effects of ginsenoside compound K on lipopolysaccharide-stimulated inflammatory responses in macrophages by regulating sirtuin 1 and histone deacetylase 4. Nutrients (2023) 15(7):1626. doi: 10.3390/nu15071626 37049466 PMC10096759

[41] Gauthier T Martin-Rodriguez O Chagué C Daoui A Ceroi A Varin A. Amelioration of experimental autoimmune encephalomyelitis by in *vivo* reprogramming of macrophages using pro-resolving factors. J Neuroinflamm (2023) 20(1):307. doi: 10.1186/s12974-023-02994-5 PMC10734130 38124095

[42] Zheng M Zhu Y Wei K Pu H Peng R Xiao J. Metformin attenuates the inflammatory response via the regulation of synovial M1 macrophage in osteoarthritis. Int J Mol Sci (2023) 24(6):5355. doi: 10.3390/ijms24065355 36982442 PMC10049635

