# Supplementary figures and images for: Songorine modulates macrophage polarization and metabolic reprogramming to alleviate inflammation in osteoarthritis
Source: Front Immunol. 2024 Feb 13;15:1344949. doi: 10.3389/fimmu.2024.1344949 (PMC10896988; doi:10.3389/fimmu.2024.1344949)

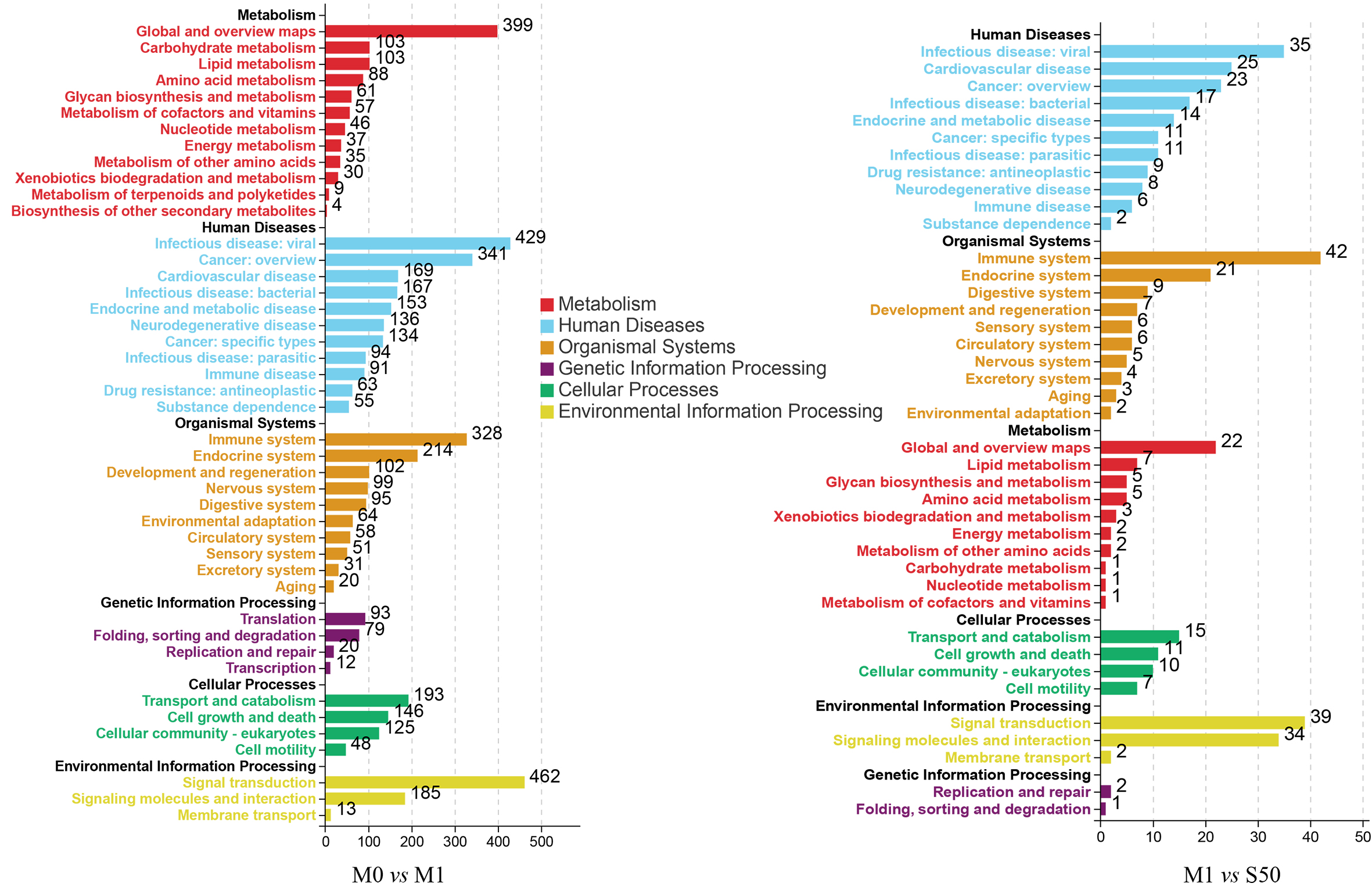

Supplement: Supplementary Figure 1 — (KEGG) database classified 44 pathways in data set 1(M0 vs M1) and 40 pathways in data set 2(M1 vs S50) into six biological process groups. [file Image_1.jpeg]

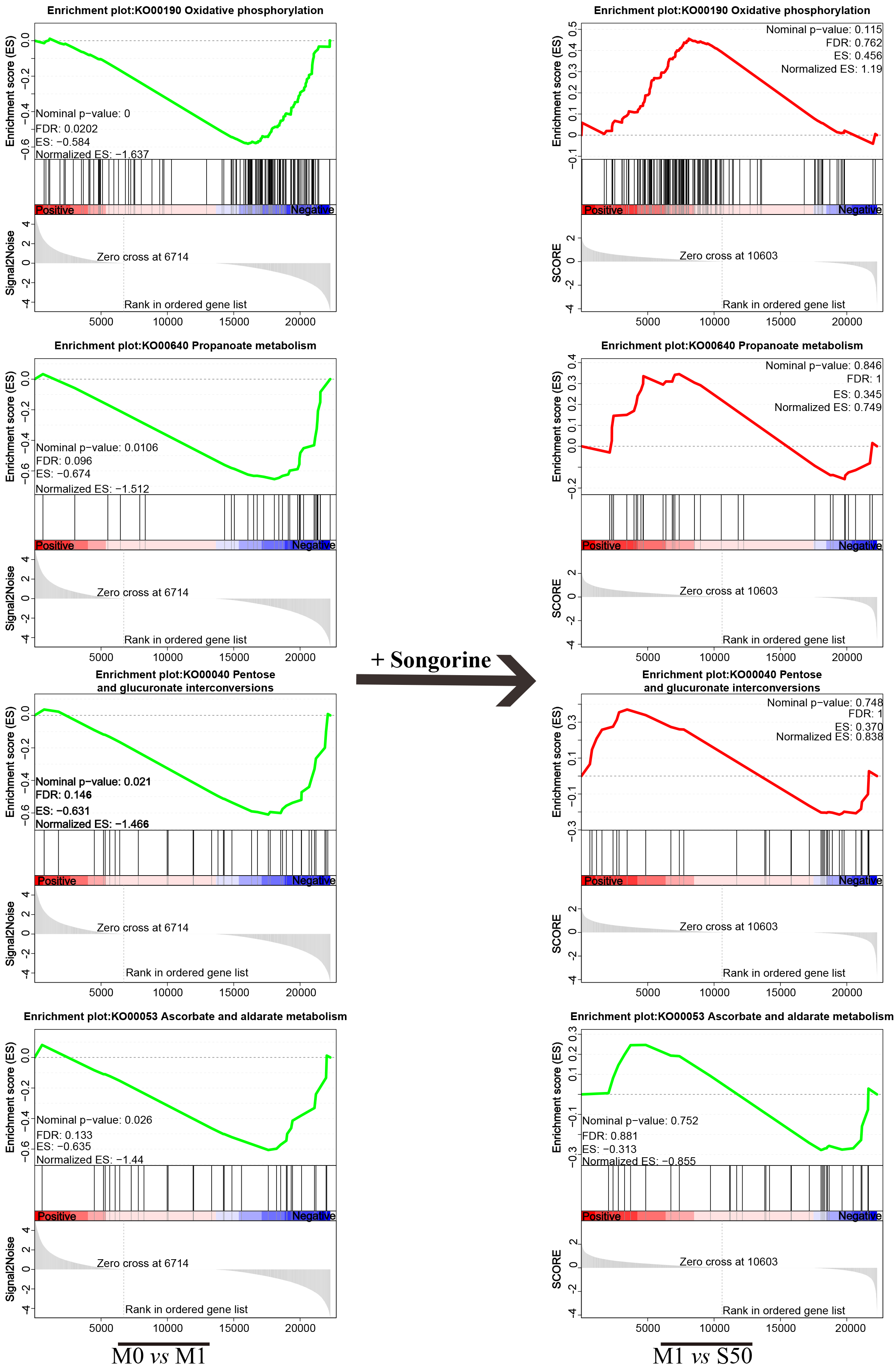

Supplement: Supplementary Figure 2 — In the GSEA analysis comparing group M1 versus group S50, Songorine was found to positively regulate glucose metabolism, correcting the metabolic dysregulation induced by LPS in macrophages. [file Image_2.jpeg]

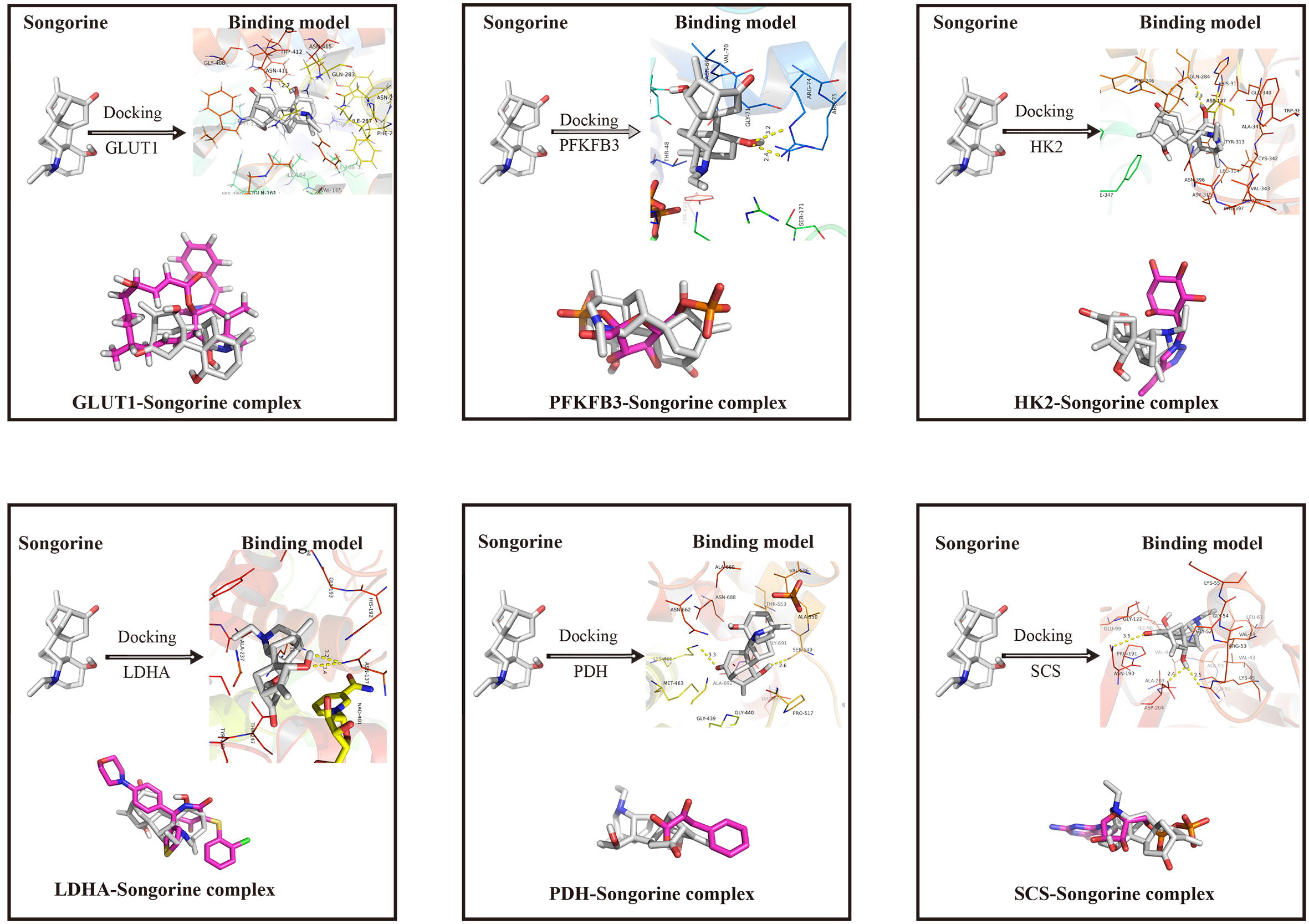

Supplement: Supplementary Figure 3 — The molecular docking diagram of Songorine binding with six metabolic targets. molecular model of Songorine (left); Three-dimensional (3D) binding model (right) and complexes (down) with the important interacting residues depicted in bright color. The backbone of the protein was rendered in tube and appears blue; Songorine is rendered silver gray; the yellow dash represents the hydrogen bond distance. [file Image_3.jpeg]

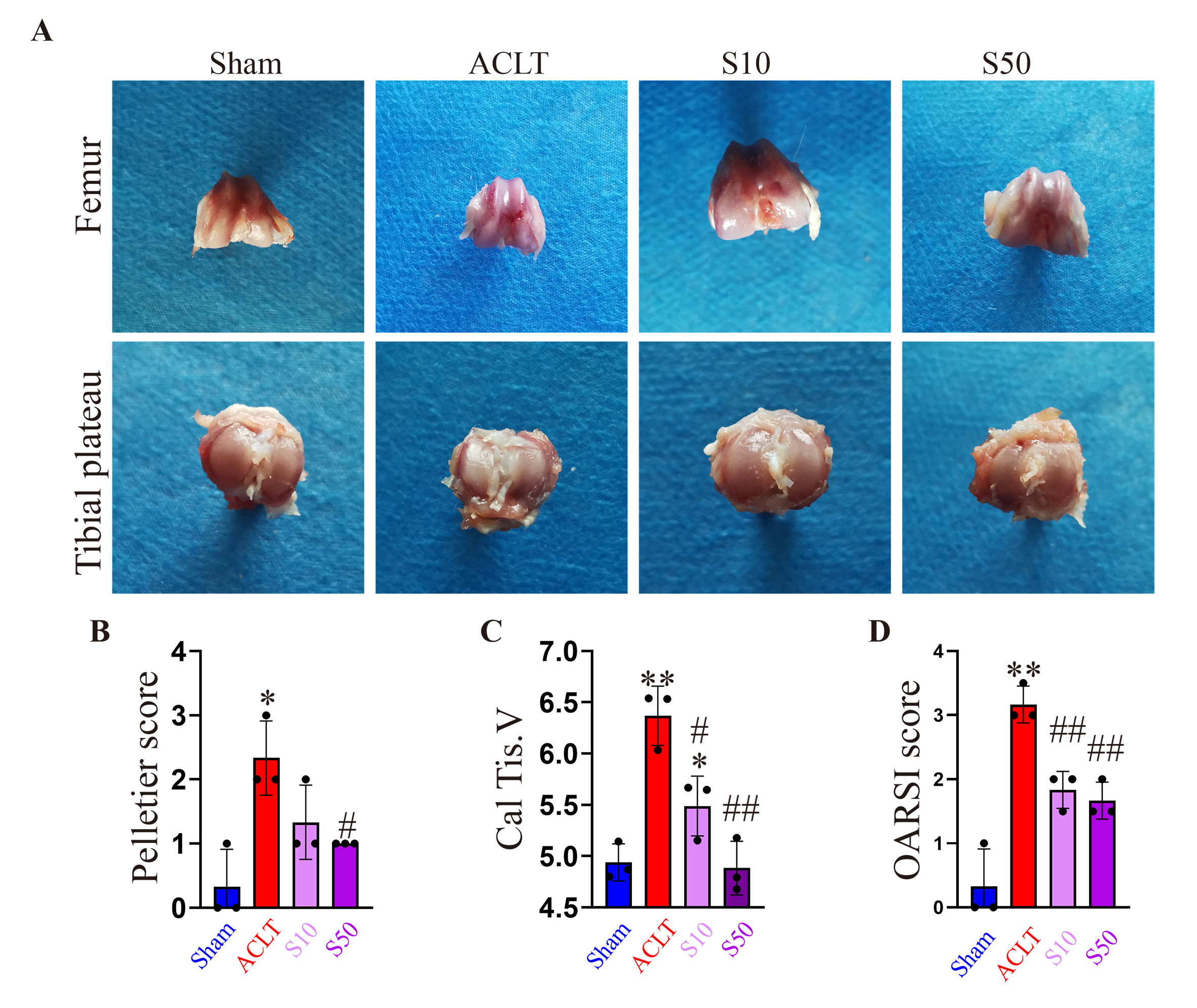

Supplement: Supplementary Figure 4 — In vivo therapeutic effect of Songorine in ACLT-Induced OA rats. (A) The gross observations of knee joint cartilage (femur and tibial plateau), the sham-operated group is marked as sham, ACLT-operated group are marked as OA, and ACLT-operated group treated with 10μM or 50μM Songorine are marked as S10 or S50. (B) The Pelletier score of knee joint cartilage. (C) Quantification of the volume of calcified meniscus and synovial tissue (Cal Tis.V). (D) Analysis of OA-like phenotype severity after ACLT surgery using the Osteoarthritis Research Society International (OARSI) score system. *p<0.05, **p<0.01, versus sham group; #p<0.05, ##p<0.01 compared with the ACLT group. [file Image_4.jpeg]

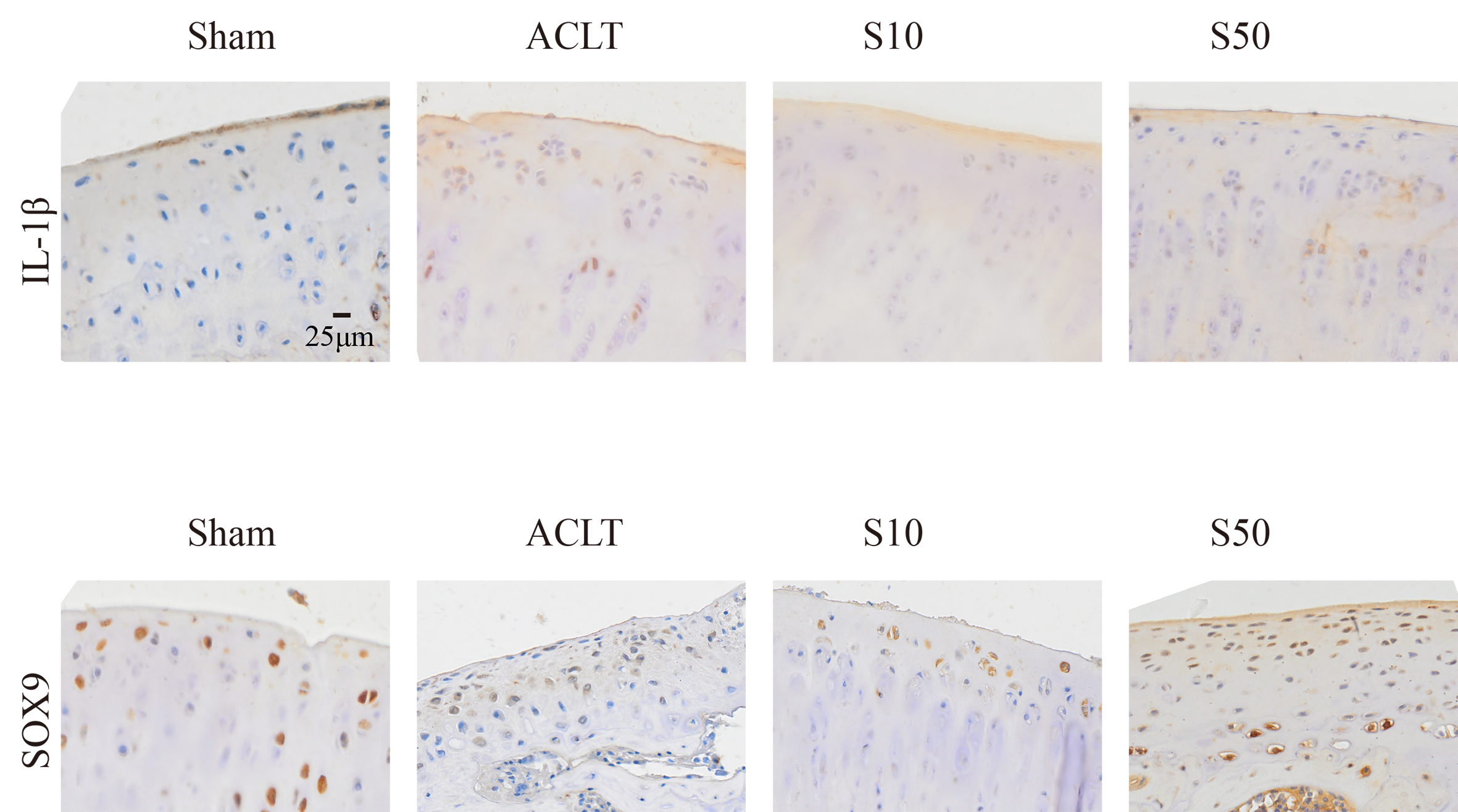

Supplement: Supplementary Figure 5 — Immunohistochemistry staining for IL-1β and SOX9 in knee joint medial compartment cartilage. Scale bar: 25μm. [file Image_5.jpeg]

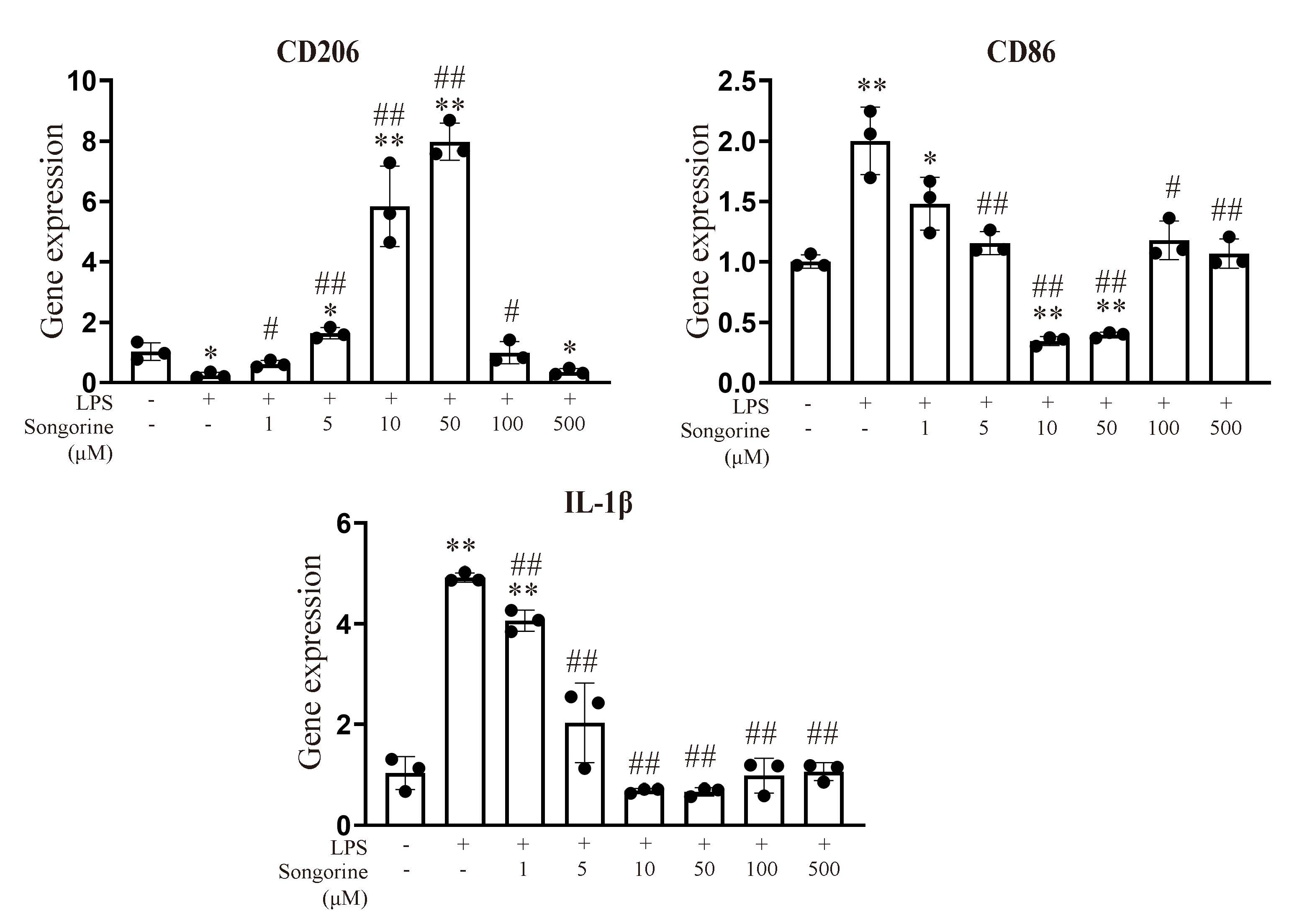

Supplement: Supplementary file 6 [file Image_6.jpeg]
